# Identification of Two *Legionella pneumophila* Effectors that Manipulate Host Phospholipids Biosynthesis

**DOI:** 10.1371/journal.ppat.1002988

**Published:** 2012-11-01

**Authors:** Ram Viner, David Chetrit, Marcelo Ehrlich, Gil Segal

**Affiliations:** 1 Department of Molecular Microbiology and Biotechnology, George S. Wise Faculty of Life Sciences, Tel-Aviv University, Tel-Aviv, Israel; 2 Department of Cell Research and Immunology, George S. Wise Faculty of Life Sciences, Tel-Aviv University, Tel-Aviv, Israel; Tufts University School of Medicine, United States of America

## Abstract

The intracellular pathogen *Legionella pneumophila* translocates a large number of effector proteins into host cells *via* the Icm/Dot type-IVB secretion system. Some of these effectors were shown to cause lethal effect on yeast growth. Here we characterized one such effector (LecE) and identified yeast suppressors that reduced its lethal effect. The LecE lethal effect was found to be suppressed by the over expression of the yeast protein Dgk1 a diacylglycerol (DAG) kinase enzyme and by a deletion of the gene encoding for Pah1 a phosphatidic acid (PA) phosphatase that counteracts the activity of Dgk1. Genetic analysis using yeast deletion mutants, strains expressing relevant yeast genes and point mutations constructed in the Dgk1 and Pah1 conserved domains indicated that LecE functions similarly to the Nem1-Spo7 phosphatase complex that activates Pah1 in yeast. In addition, by using relevant yeast genetic backgrounds we examined several *L. pneumophila* effectors expected to be involved in phospholipids biosynthesis and identified an effector (LpdA) that contains a phospholipase-D (PLD) domain which caused lethal effect only in a *dgk*1 deletion mutant of yeast. Additionally, LpdA was found to enhance the lethal effect of LecE in yeast cells, a phenomenon which was found to be dependent on its PLD activity. Furthermore, to determine whether LecE and LpdA affect the levels or distribution of DAG and PA *in-vivo* in mammalian cells, we utilized fluorescent DAG and PA biosensors and validated the notion that LecE and LpdA affect the *in-vivo* levels and distribution of DAG and PA, respectively. Finally, we examined the intracellular localization of both LecE and LpdA in human macrophages during *L. pneumophila* infection and found that both effectors are localized to the bacterial phagosome. Our results suggest that *L. pneumophila* utilize at least two effectors to manipulate important steps in phospholipids biosynthesis.

## Author Summary

*Legionella pneumophila* is an intracellular pathogen that causes a severe pneumonia known as Legionnaires' disease. Following infection, the bacteria use a Type-IVB secretion system to translocate multiple effector proteins into macrophages and generate the *Legionella*-containing vacuole (LCV). The formation of the LCV involves the recruitment of specific bacterial effectors and host cell factors to the LCV as well as changes in its lipids composition. By screening *L. pneumophila* effectors for yeast growth inhibition, we have identified an effector, named LecE, that strongly inhibits yeast growth. By using yeast genetic tools, we found that LecE activates the yeast lipin homolog – Pah1, an enzyme that catalyzes the conversion of diacylglycerol to phosphatidic acid, these two molecules function as bioactive lipid signaling molecules in eukaryotic cells. In addition, by using yeast deletion mutants in genes relevant to lipids biosynthesis, we have identified another effector, named LpdA, which function as a phospholipase-D enzyme. Both effectors were found to be localized to the LCV during infection. Our results reveal a possible mechanism by which an intravacuolar pathogen might change the lipid composition of the vacuole in which it resides, a process that might lead to the recruitment of specific bacterial and host cell factors to the vacoule.

## Introduction

*Legionella pneumophila*, the causative agent of Legionnaires' disease, is an aerobic Gram-negative pathogen that multiplies intracellularly in human phagocytic cells and in freshwater protozoa [1], [2]. The bacteria enter the cells by phagocytosis and reside within a unique phagosome, known as the *Legionella* containing vacuole (LCV), that grows in size and changes its membrane lipids composition during infection [3]. During the onset of infection, the LCV does not fuse with the host cell lysosomes nor become acidic, but instead the bacteria actively recruit secretory vesicles to the LCV and establish a replication niche [4], [5]. For the formation of the LCV, the bacteria utilize the Icm/Dot type IVB secretion system by which they translocate effector proteins that manipulate host cell processes during infection (for reviews see [6], [7]). A very similar Icm/Dot type IVB secretion system was also found in the obligate intracellular pathogen *Coxiella burnetii*, the etiological agent of Q-fever [8]–[11]. Similar to *L. pneumophila*, the Icm/Dot secretion system of *C. burnetii* was shown to be required for intracellular growth [8]. However, the intracellular lifestyle of these two pathogens is completely different [12], [13].

Currently, about 300 Icm/Dot dependent effectors have been identified in *L. pneumophila* [6] using a variety of bioinformatics and genetic screens [14]–[19]. Several of the effectors were shown to influence different host cell processes, and some of these processes are targeted by several effectors (for reviews see [7], [20]). Six effectors were found to subvert host cell vesicular trafficking by manipulating the host small GTPase Rab1: SidM/DrrA was shown to recruit Rab1 to the LCV and it activates Rab1 by functioning both as a Rab1-GEF (GDP/GTP exchange factor) and as a Rab1-GDF (GDI [GDP dissociation inhibitor] displacement factor) [21], [22]. SidM/DrrA was also shown to AMPylate Rab1 thus keeping it in its active state on the LCV [23], and the effector SidD was shown to deAMPylate Rab1 and to counteract the AMPylation of SidM/DrrA [24]. In addition, AnkX was shown to phosphocholinate Rab1, thus keeping it in its active state on the LCV [25], and Lem3 was found to dephosphocholinate Rab1 and to counteract the phosphocholination mediated by AnkX [26], [27]. An additional *L. pneumophila* effector, LidA was reported to bind Rab1 and render it active when bound to GDP or GTP [28], [29] and to tether endoplasmic reticulum (ER) derived vesicles to the LCV [30], while the effector LepB was shown to inactivate Rab1 by functioning as a Rab1-GAP (GTPase activating protein) [22]. Three *L. pneumophila* effectors (LubX, AnkB and LegU1) have been shown to be involved in ubiquitination of host cell proteins; LubX possesses two eukaryotic U-box domains and it was shown to ubiquitinate the host cell cycle protein Clk1 and the *L. pneumophila* effector SidH [17], [31]. AnkB possess a eukaryotic F-box domain and it was shown to functionally mimic eukaryotic F-box containing proteins and it exploit the host ubiquitination machinery *via* the conserved eukaryotic processes of K48-linked polyubiquitination and the proteasome machineries in order to generate free amino-acids for the bacteria [32]–[36]. LegU1 was also shown to mediate the ubiquitination of the host chaperone protein BAT3 involved in the regulation of the ER stress response [37]. Five other *L. pneumophila* effectors, including Lgt1/2/3, SidI and SidL were shown to target the host translational machinery and block protein synthesis [38]–[40] and two additional effectors, LegK1 and LnaB, were shown to activate the host cell NF-kB pathway [41], [42]. These observations clearly indicate that important host cellular processes are targeted by more than a single effector during *L. pneumophila* infection.

Beside the effectors described above, several *L. pneumophila* effectors were shown to manipulate phospholipids. Four *L. pneumophila* effectors, VipD and its paralogs VpdA, VpdB and VpdC, are homologues to phospholipase A (PLA), patatin-like, enzymes [43], [44]. PLA enzymes hydrolyze the carboxylester bonds at the carbon-1 or carbon-2 positions of phospholipids and generate fatty acids and lysophospholipids [45]. VipD was shown to possess a PLA enzymatic activity in a yeast model [44], VipD, VpdA and VpdC were reported to cause lethal effect on yeast growth when expressed, and VipD and VpdA were shown to cause secretory defects in yeast [15]. Another *L. pneumophila* effector, LegS2, was shown to act as a sphingosine-1-phosphate lyase (SPL), an enzyme that catalyze the irreversible degradation of sphingosine-1-phosphate, which is an important lipid secondary messenger, to phosphoethanolamine and hexadecanal [46]. Beside the effect on lipid composition, several *L. pneumophila* effectors (SidC, SidM/DrrA and SdcA) were shown to anchor to the LCV by specific binding to phosphatidylinositol-4 phosphate (PI4P) [47], [48], and other effectors (LidA, SetA and LpnE) were shown to preferentially bind phosphatidylinositol-3 phosphate (PI3P) [47], [49], [50].

Other bacterial pathogens have also been shown to manipulate host cell's phospholipids. Similar to *L. pneumophila*, *Salmonella enterica* resides in a unique phagosome known as the *Salmonella* containing vacuole (SCV) during infection. The *S. enterica* effector SseJ possesses a PLA and glycerophospholipid-cholesterol-acyltransferase activities. SseJ is localized to the SCV membrane where it esterifies cholesterol in order to promote infection [51], [52]. Another *S. enterica* effector involved in phospholipids manipulation is SopB (also known as SigD). SopB mediates the accumulation of PI3P on the SCV and affects multiple processes during the course of infection, including bacterial invasion, SCV formation and maturation [53]–[55]. SopB was shown to mediate PI3P accumulation by the recruitment of Rab5 to the SCV. Rab5 in-turn recruits and/or activates Vps34 which is a phosphatidylinositol (PI) 3-kinase that phosphorylates PI to produce PI3P [54]. Another example of phospholipids manipulation by a pathogen was shown in *Mycobacterium tuberculosis* which also replicates intracellularly in a phagosome [56]. The bacteria secrete the PI phosphatase SapM that specifically dephosphorylates PI3P to PI and lowers the levels of PI3P on the phagosomal membrane, thereby blocking phagosome fusion with late endosomes and lysosomes [57].

To date, *L. pneumophila* effectors were shown to be involved in the host cell's phospholipids regulation in two main aspects; i. Direct degradation of phospholipids by phospholipases (such as VipD). ii. Anchoring of effectors to the LCV *via* specific PIs (such as SidM/DrrA). In this work we present a novel strategy used by *L. pneumophila* to manipulate host cell phosphatidic acid (PA), a main component in the host cell phospholipids biosynthetic pathway. We found that the *L. pneumophila* effector LecE manipulates the PA biosynthetic pathway by activating the host PA phosphatase protein family which results in the conversion of PA to diacylglycerol (DAG). We also found that another *L. pneumophila* effector, LpdA, a phospholipase-D (PLD) enzyme, generates PA in mammalian cells and in this way it supplies additional substrate (PA) to the PA phosphatase which is activated by LecE. These findings suggest that *L. pneumophila* specifically manipulates the phospholipids composition of their phagosome to result in a successful infection.

## Results

**Figure 1:**
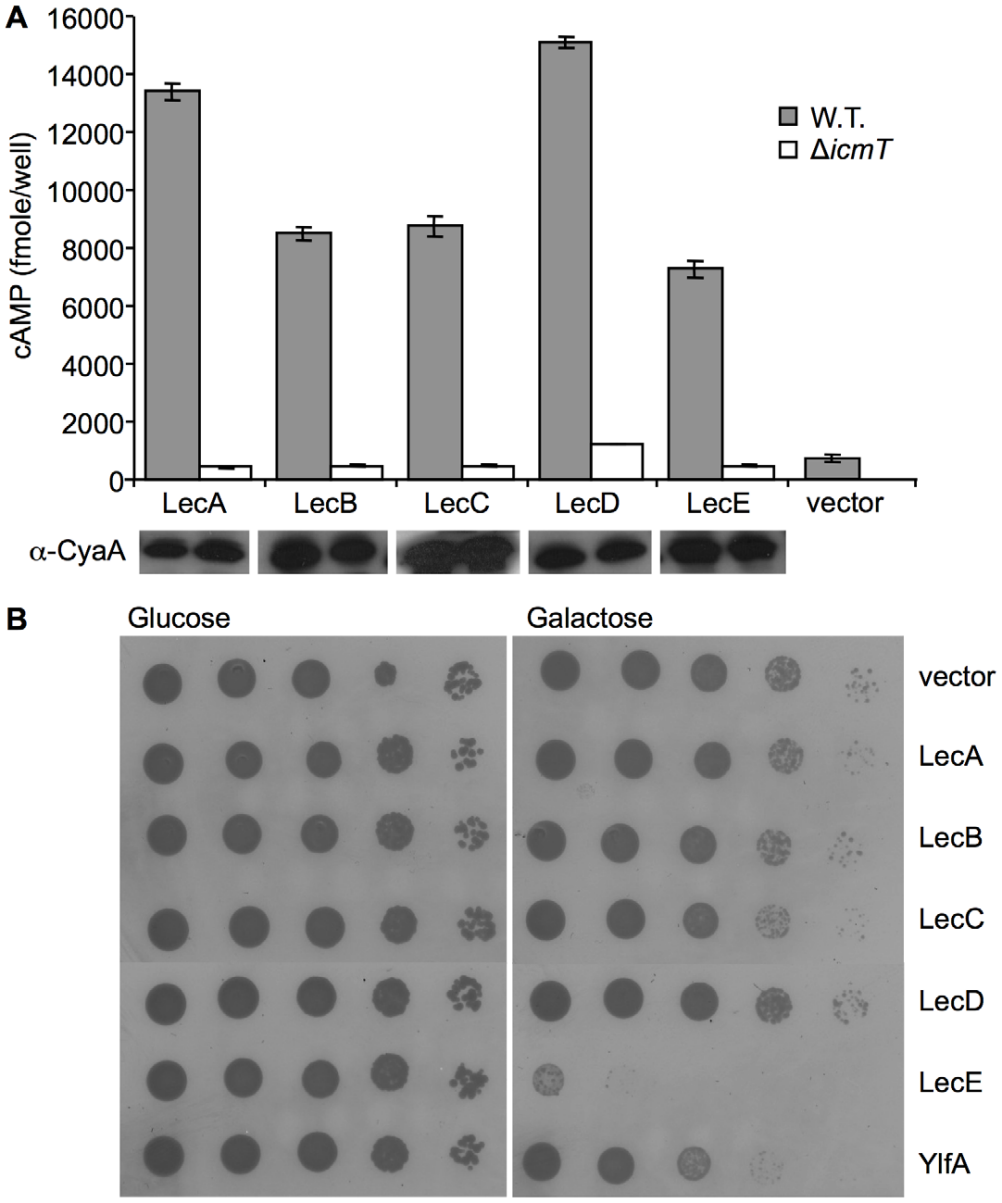
Examination of Lec effectors for translocation and inhibition of yeast growth. (A) Icm/Dot-dependent translocation of the Lec effectors. Wild-type *L. pneumophila* JR32 (gray bars) and *icm*T deletion mutant GS3011 (white bars) harboring the CyaA fusions, indicated below each bar, were used to infect HL-60-derived human macrophages and cAMP levels were determined (as described in the Materials and Methods section). The CyaA vector was used as a negative control. The protein levels of the CyaA fusions were monitored by western blot analysis using α-CyaA antibody and are presented below each bar. (B) LecE causes a strong lethal effect when over expressed in yeast. Lec effectors were cloned under the GAL1 promoter and grown on plates containing glucose or galactose in the wild-type *S. cerevisiae* BY4741. The *L. pneumophila* effector YlfA was used as a positive control. pGREG523 (vector) was used as a negative control.

*L. pneumophila* and *C. burnetii* utilize the Icm/Dot type-IVB secretion system to translocate a large number of effector proteins into host cells [6], [58], [59]. The Icm/Dot complex components of these two bacteria are conserved [9] but their intracellular infection process is completely different [7], [60]. Similarly to their intracellular lifestyle, the effector proteins translocated by these two bacteria are different and only few of them share sequence motifs such as ankyrin domains [61]. However, the high conservation of the Icm/Dot system in both bacteria in terms of sequence homology and gene organization might suggest that these bacteria also share similar effector proteins. To test this hypothesis, we performed several genomic searches aiming at the identification of proteins that show a similar phyletic distribution as the Icm/Dot proteins. We searched for genes present in the available *Legionella* and *C. burnetii* genomic sequences, discarding genes that are also present in other closely related bacteria such as *Escherichia coli* and *Pseudomonas aeruginosa* (e.g. house keeping genes). This analysis resulted with the identification of seven proteins (Table 1). To determine the involvement of these proteins in pathogenesis, we constructed CyaA fusions for the *L. pneumophila* homologs of these genes and examined them for translocation into host cells and five of them were found to encode for effector proteins (Fig. 1A). We named these proteins Lec for *Legionella* effectors with homologs in *Coxiella* (Table 1). During the course of our study, four of the *L. pneumophila* proteins (lpg1692, lpg1717, lpg2546 and lpg2552) [17], [19] and two of the *C. burnetii* proteins (CbuA0006 and Cbu_0410) [59], [62] were shown by others to translocate into host cells during infection. With the aim of identifying the function of these genes, they were cloned under the control of the galactose-regulated promoter (GAL1 promoter) and expressed in the yeast *Saccharomyces cerevisiae*. We found that LecE causes strong lethal effect on yeast growth when ectopically expressed and this lethal effect was found to be more pronounced at 37°C in comparison to 30°C (Fig. 1B and data not shown). At this point we decided to focus on LecE and to explore its function.

**Table 1:** *L. pneumophila* and *C. burnetii* homologous effector candidates.

*L. pneumophila*				*C. burnetii*		E-value	Reference or source
Lpg#	Name	Paralogs	Size	Cbu#	Name		
lpg0581		x	61	Cbu_0585		1.E-05	
lpg1692	LecA	x	436	CbuA0006	Cig66/CpeA	7.E-05	[19]
lpg1717	LecB	lpg2546	562	Cbu_1063	Cig27	3.E-12	[17]
lpg1887		x	117	Cbu_1366	Cig40	6.E-02	
lpg2164	LecC	x	154	Cbu_1950		2.E-11	This study
lpg2546	LecD	lpg1717	469	Cbu_1063	Cig27	7.E-07	[19]
lpg2552	LecE	x	555	Cbu_0410	Cig12/CoxCC3	1.E-04	[19]

### Properties of the LecE effector

**Figure 2:**
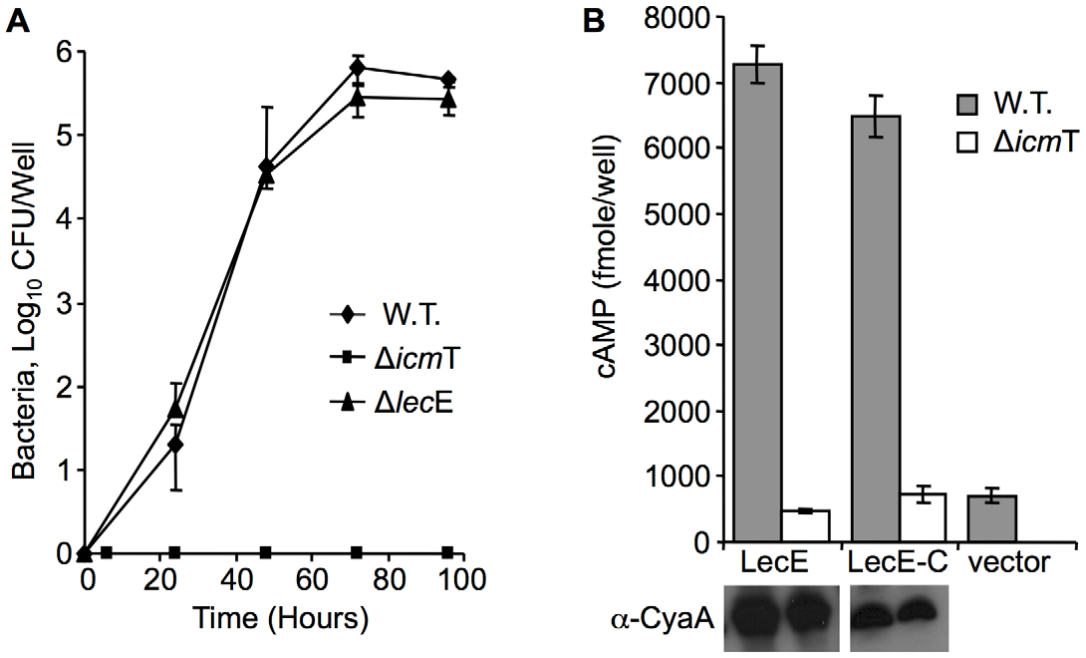
LecE contains a C-terminal translocation signal and is dispensable for intracellular growth. (A) Deletion of *lec*E from the *L. pneumophila* genome causes no intracellular growth defect in the amoebae *A. castellani*. The wild-type *L. pneumophila* JR32 (diamonds), the *lec*E deletion mutant RV-L6-45 (triangles), and the *icm*T deletion mutant GS3011 (squares) were examined. (B) LecE posses a C-terminal, Icm/Dot dependent, translocation signal. Wild-type *L. pneumophila* JR32 (gray bars) and *icm*T deletion mutant GS3011 (white bars) harboring the CyaA fusions, indicated below each bar, were used to infect HL-60-derived human macrophages and cAMP levels were determined (as described in the Materials and Methods section). LecE-C contains the 92 C-terminal amino-acids of LecE fused to CyaA. The CyaA vector was used as a negative control. The protein levels of the CyaA fusions were monitored by western blot analysis using α-CyaA antibody and are presented below each bar.

The LecE protein (Lpg2552) is 555 amino acids long, and is predicted to contain at least six hydrophobic domains which are most likely associated with membranes after translocation into host cells. To examine the involvement of LecE in *L. pneumophila* intracellular growth we constructed a deletion substitution mutant in the gene encoding for LecE and examined it for intracellular growth in *Acanthamoeba castellanii* and HL-60 derived human macrophage. Similarly to most of the *L. pneumophila* effectors, the deletion of *lec*E had no effect on the intracellular growth in both hosts (Fig. 2A, and data not shown). In addition, similarly to other *L. pneumophila* effectors, the translocation signal of LecE was found to be located at the C-terminus, since a CyaA fusion of the 92 C-terminal amino acids of LecE was found to translocate into host cells with a similar efficiency like the full length protein (Fig. 2B).

### Identification of yeast suppressors for the LecE lethal effect

**Figure 3:**
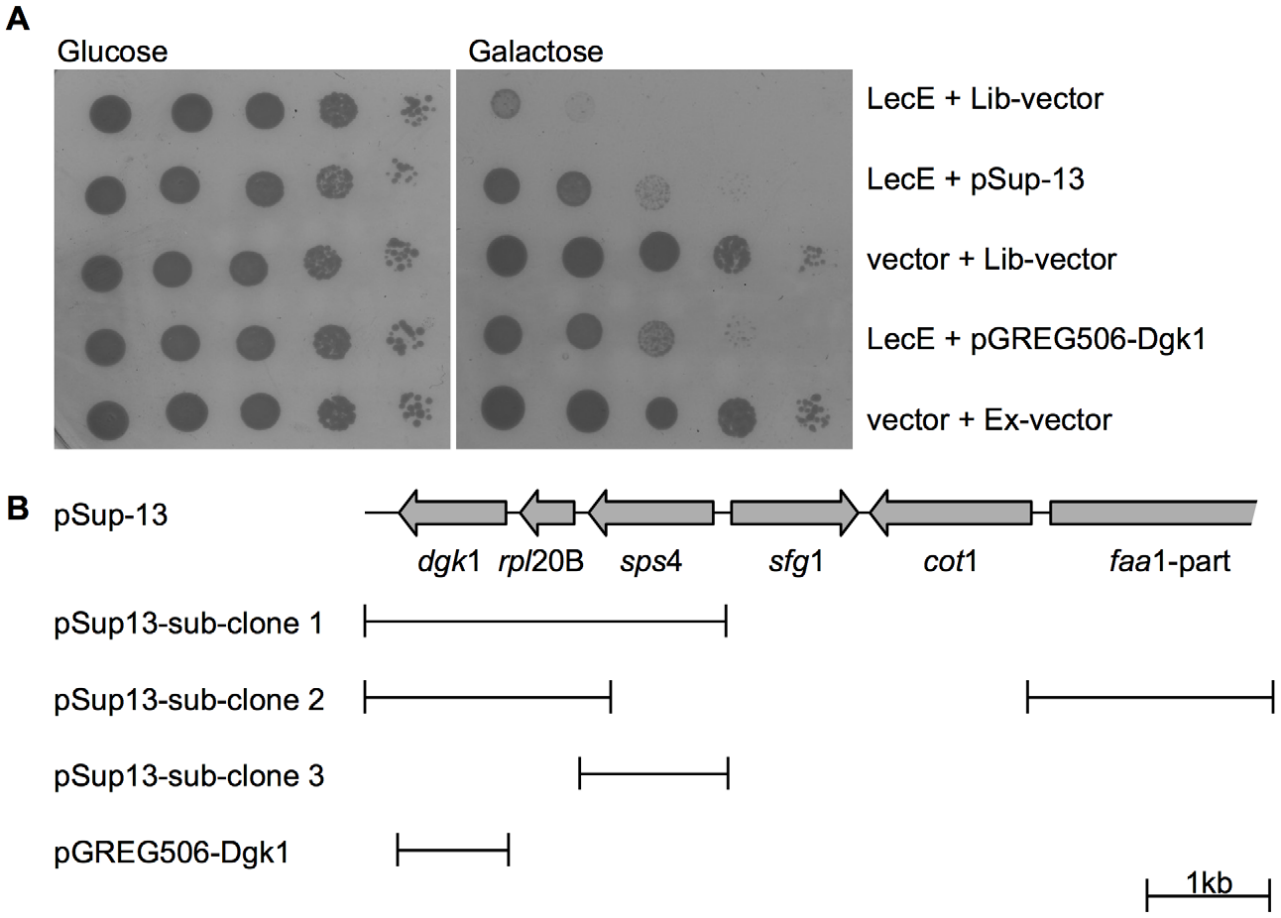
Over expression of the yeast Dgk1 protein suppressed the LecE lethal effect on yeast growth. (A) LecE was over expressed in wild-type *S. cerevisiae* BY4741 together with the library vector pYep24 (Lib-vector), the original suppressor plasmid (pSup-13), the *dgk*1 gene cloned under the GAL1 promoter (pGREG506-Dgk1) or the over expression vector pGERG506 (Ex-vector). LecE was expressed from pGREG523 (vector). (B) Diagram of pSup-13 genomic fragment and its sub-clones. The genes are presented by grey arrows. The DNA fragment present in each sub-clone is indicated by lines under the diagram of pSup-13.

To identify the cellular target of LecE we decided to use a *S. cerevisiae* high-copy number genomic library, and look for colonies that grow in the presence of LecE, at 37°C, under inducing conditions (media supplemented with galactose). Several colonies where isolated (see Materials and Methods), and most of them did not produce a full-length LecE (data not shown), however one suppressor colony produced a full length LecE protein and the yeast cells were able to grow under LecE inducing conditions (Sup13 in Fig. 3A). The library plasmid present in this suppressor colony was isolated and reintroduced into a yeast strain containing the galactose inducible *lec*E gene and similar suppression was obtained. Sequencing of the two edges of the plasmid insert revealed the genomic region responsible for the suppression observed (Fig. 3B). Several subclones that were constructed (Fig. 3B) indicated that the *dgk*1 gene is the gene responsible for the suppression effect. To further confirm the results obtained, we cloned the *dgk*1 gene under the GAL1 promoter and both *lec*E and *dgk*1 containing plasmids were introduced into yeast. As can be seen in Fig. 3A, Dgk1 over expression showed clear suppression of the lethal effect caused by LecE.

**Figure 4:**
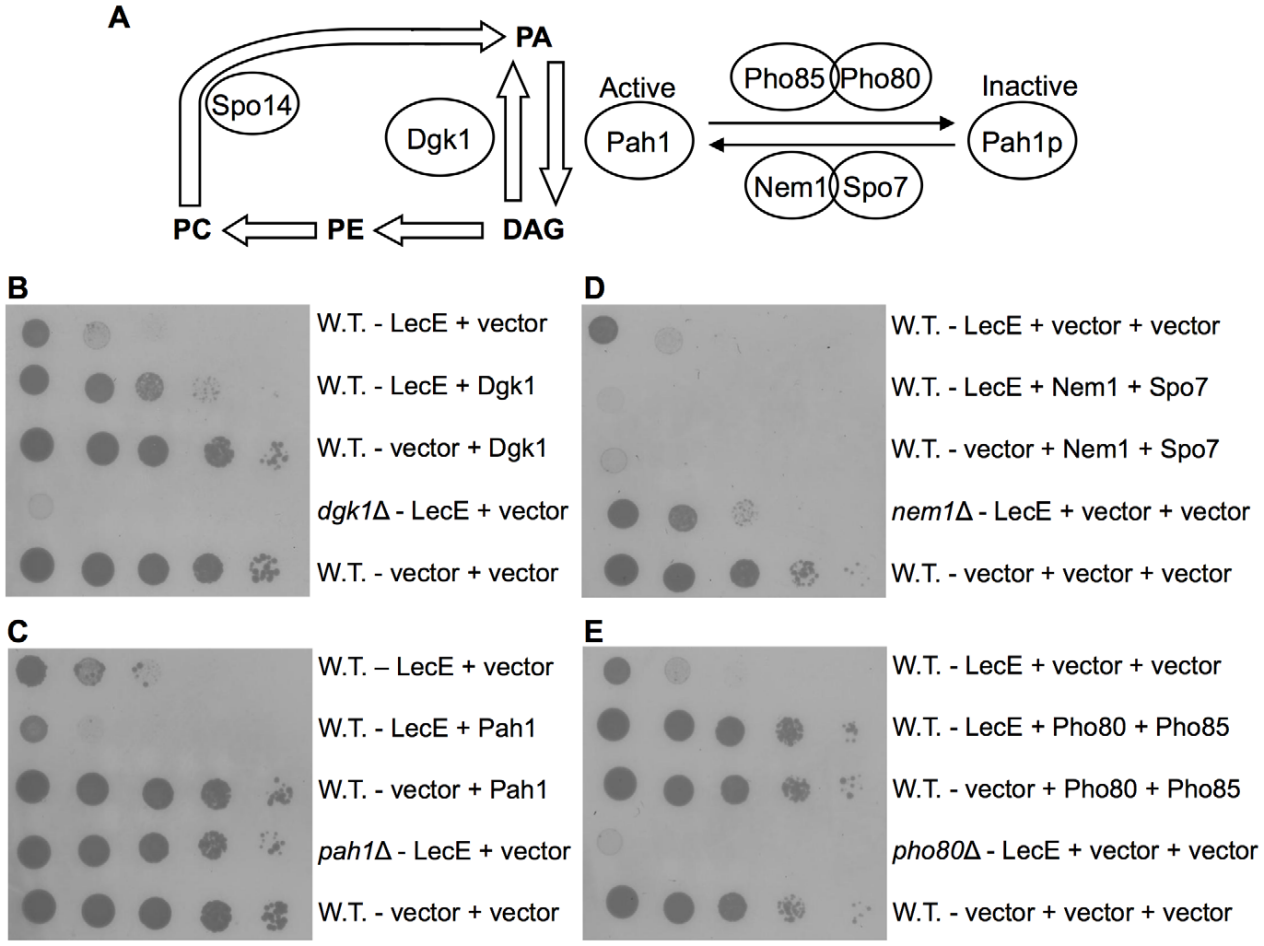
The yeast gene *pah*1 is required for LecE's lethal effect on yeast growth. (A) The Phosphatidic acid - Diacylglycerol biosynthetic pathway in yeast. Lipid compounds are marked in uppercase letters and bold case. Enzymes known to catalyze individual steps in the phospholipids biosynthesis pathways in yeast are indicated in circles. PA, phosphatidic acid; DAG, diacylglycerol; PE, phosphatidylethanolamine; PC, phosphatidylcholine. Dgk1 is a DAG-kinase, Spo14 is a phospholipase D (PLD) and Pah1 is a PA-phosphatase. Pah1p is the inactive form of Pah1 and its activation is mediated by the Nem1-Spo7 phosphatase complex while its inactivation is mediated by the Pho85-Pho80 kinase-cyclin complex. (B, C, D, E) Comparison of the LecE lethal effect on different yeast deletion mutants and strains over-expressing yeast genes involved in phospholipids biosynthesis. (B) LecE lethal effect on yeast growth was enhanced in a *dgk*1 deletion mutant. LecE was over-expressed in a wild-type *S. cerevisiae* BY4741 (W.T.) together with Dgk1 cloned under the GAL1 promoter or in the *dgk*1 deletion mutant (*dgk1*Δ). The LecE and Dgk1 vectors were pGREG523 (vector) and pGREG506 (vector), respectively. (C) Deletion of *pah*1 suppressed the lethal effect of LecE on yeast growth. LecE was over-expressed in a wild-type *S. cerevisiae* BY4741 (W.T.) together with Pah1 cloned under the GAL1 promoter or in the *pah*1 deletion mutant RV-L8-59 (*pah1*Δ). The LecE and Pah1 vectors were pGREG523 (vector) and pGREG505 (vector), respectively. (D) Deletion of *nem*1 partially suppressed the lethal effect of LecE on yeast growth. LecE was over-expressed in a wild-type *S. cerevisiae* BY4741 (W.T.) together with Nem1 and Spo7 or in the *nem*1 deletion mutant RV-L8-54 (*nem1*Δ). The LecE, Nem1 and Spo7 vectors were pGREG523 (vector), pGREG506 (vector) and pGREG505 (vector), respectively. (E) Over expression of the Pho85-Pho80 kinase-cyclin complex completely suppressed the LecE lethal effect on yeast growth. LecE was over-expressed in a wild-type *S. cerevisiae* BY4741 (W.T.) together with Pho85 and Pho80 or in the *pho*80 deletion mutant (*pho80*Δ). The LecE, Pho85 and Pho80 vectors were pGREG523 (vector), pGREG506 (vector) and pGREG505 (vector), respectively. The glucose control plates are presented in Fig. S1.

Dgk1 is a diacylglycerol-kinase enzyme that catalyzes the formation of phosphatidic acid (PA) from diacylglycerol (DAG) and counteracts the phosphatase activity of the enzyme Pah1 on PA (Fig. 4A) [63]. The activity of Pah1 has been shown to be dependent on its phosphorylation state, and it was shown to be active when de-phosphorylated [64]. The kinase-cyclin complex Pho85-Pho80 has been shown to phosphorylate Pah1 thus inactivating it [65] and the Nem1-Spo7 phosphatase complex has been shown to dephosphorylate Pah1 and activate it [64]. It is important to note that over expression of Dgk1 was found before as a single suppressor in two screens: i) In a screen aimed at identifying yeast suppressors that can rescue the lethal effect caused by the over expression of Pah1-7P (a constitutively dephosphorylated and therefore active Pah1) [63] and ii) In a screen aimed at identifying yeast suppressors that can rescue the lethal effect caused by the over expression of the yeast Nem1-Spo7 phosphatase complex that dephosphorylates and therefore activates Pah1 [63]. In both screens, Dgk1 over expression suppresses a highly active Pah1 enzyme, what might indicate that this is also the outcome of the over expression of the *L. pneumophila* effector LecE.

### Analysis of the relationships between LecE and the yeast Dgk1 and Pah1 proteins

The Dgk1 suppression of the LecE lethal effect can be explained in several ways: i) LecE might inhibit the function of Dgk1, in this case higher levels of Dgk1 will result in some Dgk1 that will be left active in the cells; ii) LecE might directly activate the function of Pah1, in this case higher levels of Dgk1, which performs the opposite enzymatic reaction, will suppress the effect of Pah1 activation by LecE. There are also two indirect ways by which Pah1 might be activated by LecE: iii) LecE might activate the Nem1-Spo7 phosphatase complex, that activates Pah1, and in this way it might activate Pah1 indirectly, and iv) LecE might inhibit the Pah1 kinase-cyclin complex Pho85-Pho80 that inactivates Pah1 and in this way it might activate Pah1 indirectly. v) An additional possibility might be that LecE itself possesses an enzymatic activity like Pah1 (PA phosphatase) and Dgk1 suppresses the effect of LecE simply because it performs the opposite enzymatic reaction. To sort between these possibilities we used several yeast deletion mutants and strains over expressing relevant yeast genes and the results of these analyses are presented in Fig. 4B, C, D, E and Fig. S1.

If LecE inhibits the function of Dgk1 then we would expect that a deletion mutant in *dgk*1 will be lethal to yeast, however it is known that a deletion in *dgk*1 is viable and show no yeast growth defects ([66] and Fig. 4B). In addition, when we over expressed LecE in the *dgk*1 deletion strain the lethal effect of LecE was even stronger in comparison to the effect on wild-type yeast (Fig. 4B) indicating that LecE causes its lethal effect also in the absence of Dgk1, therefore it is not possible that the lethal effect observed in the wild-type strain occurred due to inhibition of Dgk1 activity. Moreover, the result showing that LecE caused a stronger lethal effect in the *dgk*1 deletion mutant, in comparison to its lethal effect in the wild-type yeast (Fig. 4B), supports the possibility that LecE activates the opposite reaction which is catalyzed by Pah1. If LecE activates the function of Pah1 then its expression in a *pah*1 deletion mutant is expected to result with suppression of the LecE lethal effect because its target protein will be missing. Thus, LecE was over expressed in a *pah*1 deletion mutant and the result obtained was very clear, the deletion in the gene encoding for *pah*1 almost completely eliminated the lethal effect of LecE (Fig. 4C), clearly showing that Pah1 is required in order for LecE to cause its lethal effect on yeast growth. In addition, when LecE and Pah1 were over expressed together the lethal effect of LecE was enhanced, even though Pah1 by itself had no effect on yeast growth (Fig. 4C). The combined results indicate that Pah1 is activated by LecE and that this activation causes the observed LecE lethal effect on yeast growth. The fact that Pah1 was required for LecE to cause its lethal effect on yeast growth also indicates that *lec*E does not encode for a PA phosphatase enzyme by itself (the Pah1 activity) since in this case the deletion in *pah*1 should have had no effect on the lethal effect caused by LecE.

In order to test whether LecE directly activates the Pah1 function or indirectly by targeting the Pah1 regulators, the relations between LecE and the Nem1-Spo7 phosphatase complex that activates Pah1 and the kinase-cyclin complex Pho85-Pho80 that inactivates Pah1, were examined. To examine if LecE activates the Nem1-Spo7 phosphatase complex, LecE was over expressed in the *nem*1 deletion mutant (*nem*1 encodes for the catalytic subunit of the phosphatase complex) or together with the Nem1-Spo7 phosphatase complex. As shown in Fig. 4D, a deletion in *nem*1 weakly suppressed the lethal effect caused by LecE while the over expression of LecE together with the Nem1-Spo7 phosphatase complex enhanced the lethal effect compared to LecE or the phosphatase complex by themselves. Both results indicate that the Nem1-Spo7 phosphatase complex is not targeted by LecE, but that both LecE and the Nem1-Spo7 phosphatase complex perform a similar function that results with the activation of Pah1 (see below). According to this hypothesis, when *nem*1 is missing some of the Pah1 protein remains inactive and therefore a weak suppression effect was observed, while when LecE was over expressed together with the Nem1-Spo7 phosphatase complex they both activate Pah1, resulting with an enhanced lethal effect. An additional way for LecE to indirectly activate Pah1 is to inhibit the function of the Pah1 kinase-cyclin complex Pho85-Pho80 that was shown to phosphorylate Pah1 and in this way inactivate it [65]. To examine this possibility, LecE was over expressed together with the Pho85-Pho80 kinase-cyclin complex or in the *pho*80 deletion mutant. As shown in Fig. 4E, the over expression of the Pho85-Pho80 kinase-cyclin complex completely suppressed the LecE lethal effect on yeast growth while in the *pho*80 deletion mutant the lethal effect of LecE was enhanced (comparable results were obtained when LecE was over expressed in the *pho*85 deletion mutant, data not shown). The enhanced lethality of LecE in the *pho*80 and *pho*85 deletion mutants indicates that the Pho85-Pho80 kinase-cyclin complex is not targeted by LecE. In addition, the suppression of the LecE lethal effect by the Pho85-Pho80 kinase-cyclin complex indicates that its function is opposite to the one of LecE. In conclusion, the analyses performed in the yeast system strongly indicate that LecE directly activates Pah1.

### The lethal effect of LecE or the Nem1-Spo7 complex is suppressed by the same yeast genetic backgrounds

**Figure 5:**
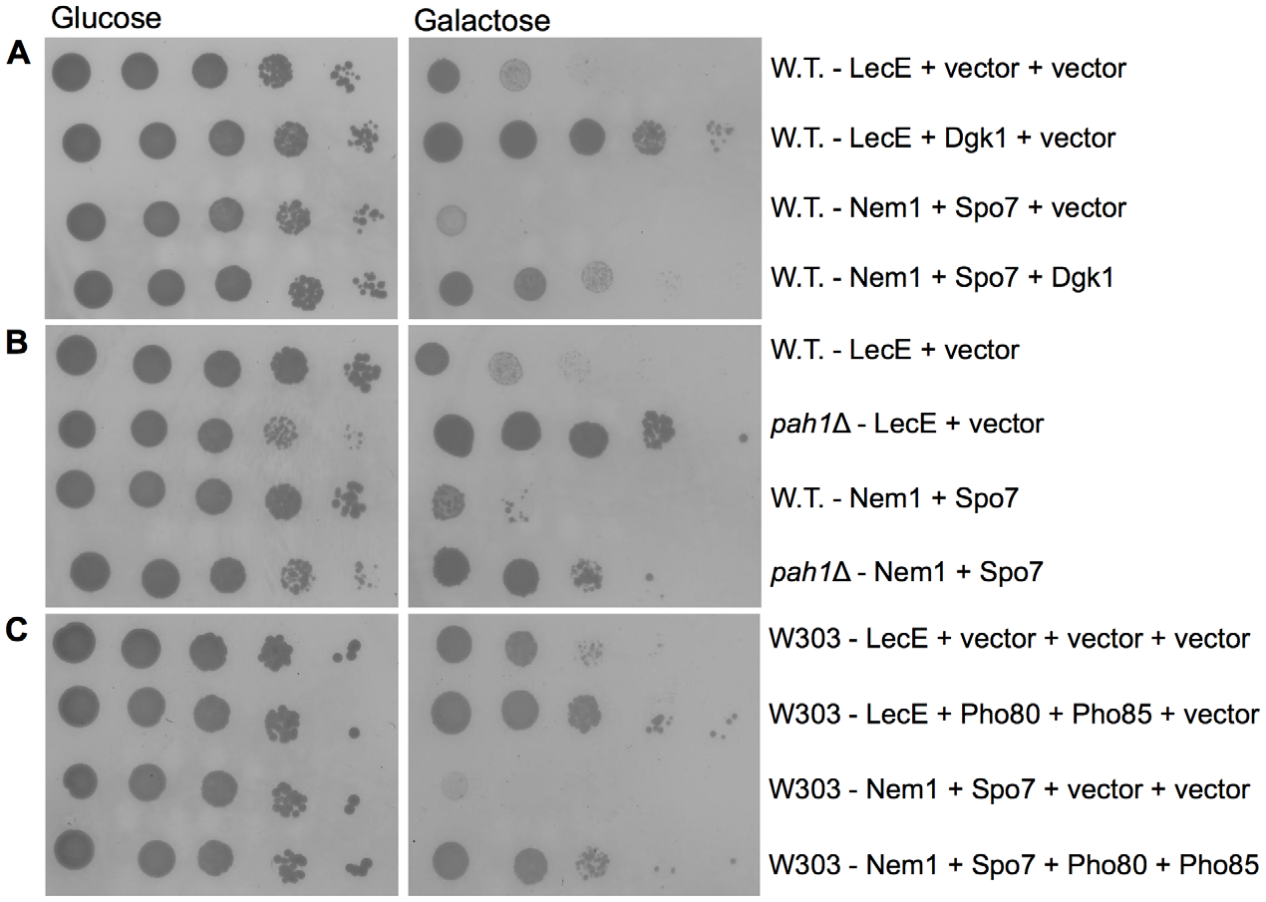
The LecE and the Nem1-Spo7 phosphatase complex lethal effect on yeast growth are suppressed by the same yeast genetic backgrounds. (A) Over expression of Dgk1 suppresses the lethal effect caused by the over expression of the Nem1-Spo7 phosphatase complex in yeast, similarly to LecE. LecE (upper two rows) or Nem1 and Spo7 (lower two rows) were over expressed in wild-type *S. cerevisiae* BY4741 (W.T.) together with Dgk1 or with pGREG506 (vector). (B) Deletion of *pah*1 suppressed the lethal effect caused by the over expression of the Nem1-Spo7 phosphatase complex in yeast, similarly to LecE. LecE (upper two rows) or Nem1 and Spo7 (lower two rows) were over expressed in wild-type *S. cerevisiae* or in the *pah*1 deletion mutant RV-L8-59 (*pah1*Δ). (C) Over-expression of the Pho85-Pho80 kinase-cyclin complex suppresses the lethal effect caused by the over expression of the Nem1-Spo7 phosphatase complex in yeast, similarly to LecE. LecE (upper two rows) or Nem1 and Spo7 (lower two rows) were over-expressed in wild-type *S. cerevisiae* W303 (W303) together with Pho85 and Ph080 or with the relevant vectors. The experiment was preformed at 30°C.

To further validate the possibility that LecE functions similarly to the Nem1-Spo7 phosphatase complex, we directly compared the effect of LecE and the Nem1-Spo7 phosphatase complex on yeast growth (Fig. 5). We found that over expression of LecE or the Nem1-Spo7 phosphatase complex are both lethal to yeast growth and their lethal effect was suppressed by over expression of Dgk1 (Fig. 5A) and by a deletion of the gene encoding for Pah1 (Fig. 5B). In addition, to test whether the Pho85-Pho80 kinase-cyclin complex also suppresses the lethal effect of the Nem1-Spo7 phosphatase complex on yeast growth, a different yeast strain (W303) that allowed the introduction of four plasmids, was used. Since this strain grows slowly at 37°C it was incubated at 30°C where the LecE lethal effect was less pronounced (see above). Similarly to the over expression of Dgk1 and the deletion of *pah*1, the over expression of the Pho85-Pho80 kinase-cyclin complex also suppressed the lethal effect on yeast growth of both LecE and the Nem1-Spo7 phosphatase complex (Fig. 5C). These results indicate that LecE directly activates Pah1 similarly to the Nem1-Spo7 phosphatase complex.

### The enzymatic activity of Dgk1 is required for the suppression of the LecE lethal effect

**Figure 6:**
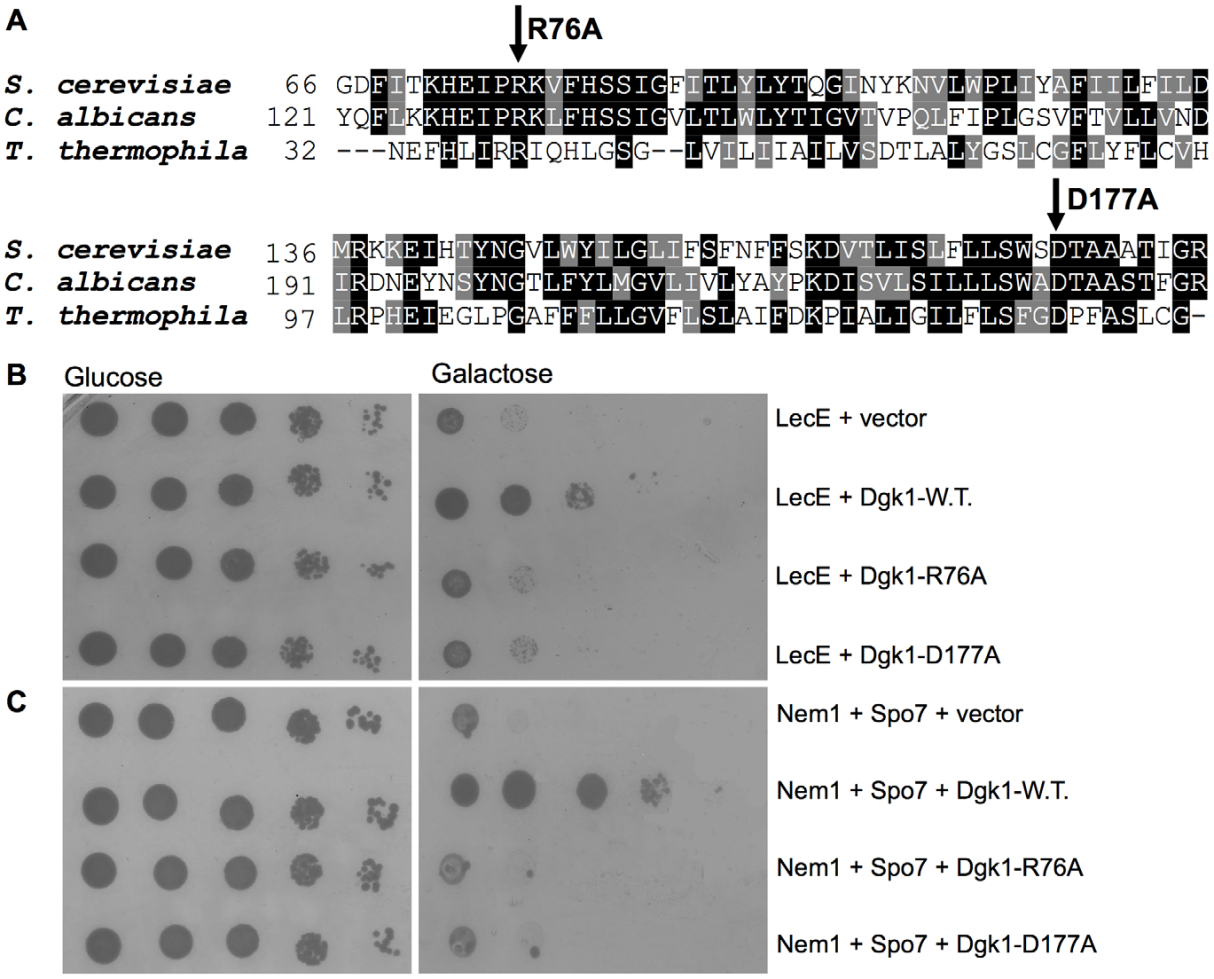
The DAG-kinase activity of Dgk1 is required for suppression of the lethal effect on yeast growth caused by LecE. (A) Part of a protein sequence alignment of *S. cerevisiae* Dgk1 and its homologs from the fungus *Candida albicans* and the ciliate protozoa *Tetrahymena thermophila*. The amino-acids R76 and D177 that were mutated are marked by arrows. (B, C) Point mutations in the DAG-kinase active site of Dgk1 abolished its suppression of the lethal effect on yeast growth caused by LecE or by the Nem1-Spo7 phosphatase complex. LecE (B) or Nem1 and Spo7 (C) were over expressed in wild-type *S. cerevisiae* BY4741 together with the wild-type Dgk1 (Dgk1-W.T.), its two point mutants, Dgk1-R76A and Dgk1-D177A or pGREG506 (vector). Dgk1-R76A and Dgk1-D177A are point mutations in Dgk1 that were shown before to be required for its DAG-kinase activity [67].

As indicated above, the *S. cerevisiae dgk1* gene encodes for a DAG kinase enzyme that catalyzes the formation of PA from DAG. Unlike the DAG kinases from bacteria, plants, and animals, the yeast enzyme utilizes CTP, instead of ATP, as the phosphate donor in the reaction [67]. Point mutations of conserved residues within the Dgk1 CTP transferase domain were shown before to result in a loss of DAG kinase activity [67]. To determine if the enzymatic activity of Dgk1 is required for the suppression of the lethal effect caused by LecE, we generated two point mutations (R76A and D177A) in Dgk1 that were shown before to abolish the DAG kinase activity [67] (Fig. 6A). As can be seen in Fig. 6B, both mutated Dgk1 proteins were unable to suppress the lethal effect of LecE in comparison to the wild-type Dgk1, indicating that an enzymatically active Dgk1 is required for suppression. The same result was also obtained for the lethal effect caused by the over expression of the Nem1-Spo7 phosphatase complex (Fig. 6C), further demonstrating the similar function of LecE and the Nem1-Spo7 complex.

### The enzymatic activity of Pah1 is required for LecE to cause lethal effect on yeast growth

The *S. cerevisiae* Pah1 belongs to a highly conserved family of proteins, called lipins. This novel family of Mg^+2^-dependent PA-phosphatase enzymes catalyze a fundamental reaction in lipid biosynthesis, namely the dephosphorylation of PA to DAG. Lipins are highly conserved throughout the eukaryotic kingdom and exhibit similar overall primary organization [68]. They are relatively large proteins (close to 100 kDa) and contain a conserved amino-terminal domain (N-LIP) of unknown function, and a carboxy-terminal catalytic domain (C-LIP) harboring an invariable HAD-like phosphatase motif, the DXDXT motif [68]–[70].

**Figure 7:**
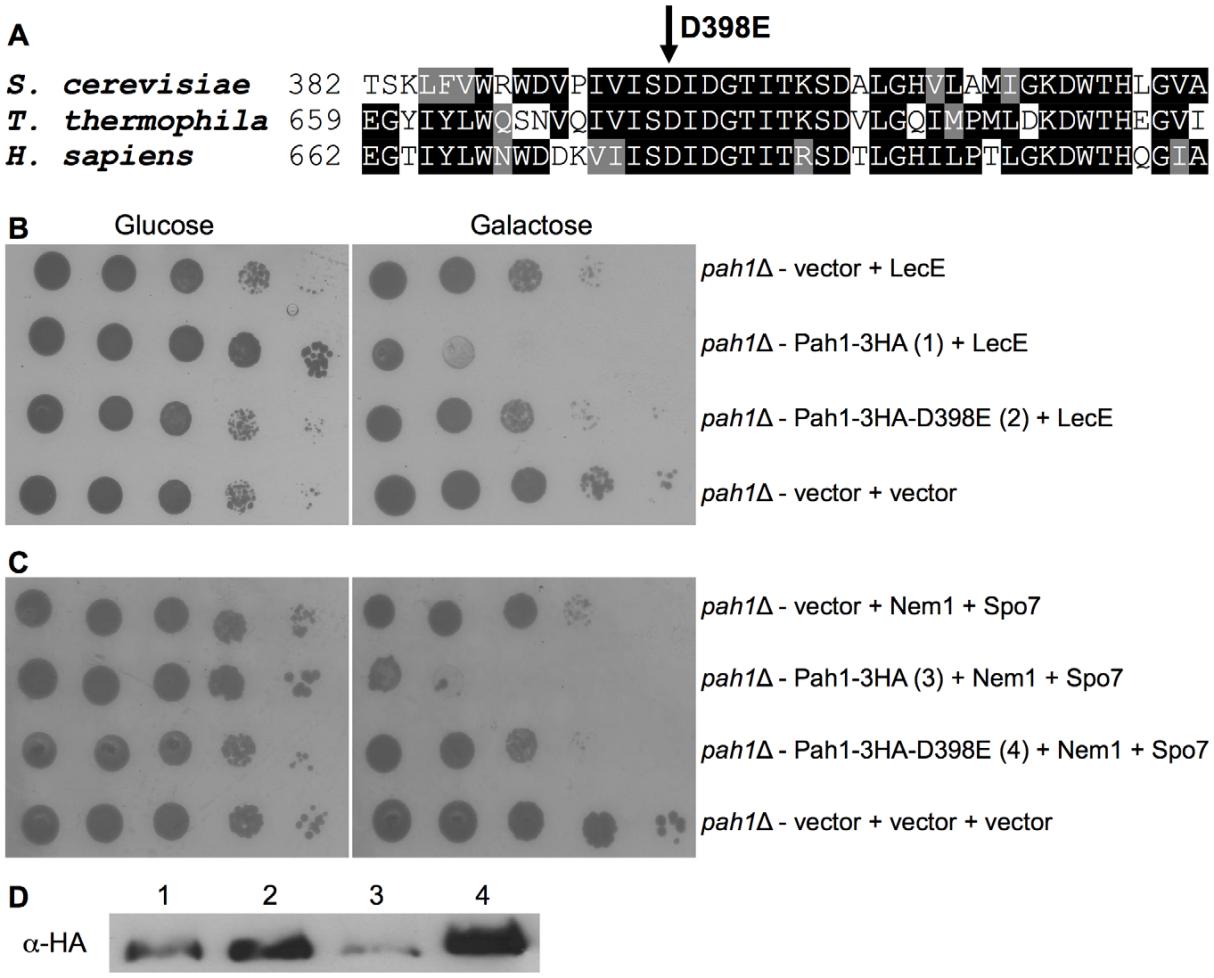
The PA-phosphatase activity of Pah1 is required for LecE in order to cause lethal effect on yeast growth. (A) Part of a protein sequence alignment of *S. cerevisiae* Pah1 and its homologs from the ciliate protozoa *T. thermophila* and *Homo sapiens* (Lipin1). The amino-acid D398 that was mutated is marked. (B, C) An HA-tagged wild-type Pah1 restores the LecE or the Nem1-Spo7 phosphatase complex lethal effect in a *pah*1 deletion mutant. LecE (B) or Nem1 and Spo7 (C) were over expressed in the *pah*1 deletion mutant RV-L8-59 (*pah1*Δ) together with the HA-tagged wild-type Pah1 (Pah1-3HA), the mutated Pah1-D398E (Pah1-3HA-D398E) or pGREG505 (vector). The Pah1 containing the D398E point mutation was shown before to completely lose its PA-phosphatase activity [71]. pGREG523 (vector), pGREG506 (vector) and pGREG503 (vector) were used as negative controls for LecE, Nem1 and Spo7, respectively. (D) The HA-tagged Pah1 protein levels were monitored by western blot analysis using α-HA antibody. The numbers correspond to these in sections B and C.

To determine if the enzymatic activity of Pah1 is required for LecE to cause its lethal effect on yeast growth, we generated a point mutation (D398E) in the conserved DXDXT motif of Pah1 (Fig. 7A) that was shown before to be critical for the PA phosphatase activity of Pah1 [71]. To determine the outcome of this mutation on yeast cells in relation to LecE, we first constructed an HA-tagged wild-type Pah1 and introduced it into yeast containing a deletion in the *pah*1 gene and LecE. The introduction of the HA-tagged Pah1 restored the lethal effect of LecE on yeast cells (Fig. 7B), however when the mutated HA-tagged Pah1 (D398E) was introduced instead of the wild-type Pah1 protein the lethal effect of LecE was not restored, indicating that an enzymatically active Pah1 is required to be present in the yeast cells in order for LecE to cause its lethal effect (both the wild-type and mutated HA-tagged Pah1 proteins were expressed in the yeast cells examined, Fig. 7D). Like in the case of the mutated Dgk1, a similar result to the one obtained with LecE was also obtained with the over expression of the Nem1-Spo7 phosphatase complex (Fig. 7C), further demonstrating the similar function of LecE and this complex.

### LecE does not function as a phosphatase of Pah1

The results described thus far, clearly demonstrate that LecE requires the presence of an enzymatically active Pah1 protein in the yeast cells in order to cause its lethal effect on yeast growth, and this requirement is identical to the one of the Nem1-Spo7 phosphatase complex. However, the mechanisms of action by which effectors activate host cell factors are often different than the ways by which these host factors are activated naturally (see Introduction). To further determine the mechanism of activation of Pah1 by LecE, we examined the size of the Pah1 protein in yeast cells over expressing the LecE effector in comparison to the over expression of the Nem1-Spo7 phosphatase complex. Western analysis showed a clear reduction in the size of the Pah1 protein when the Nem1-Spo7 phosphatase complex was over expressed in yeast but no change in the apparent molecular weight of Pah1 was observed when LecE was over expressed (Fig. S2). These results indicate that LecE activates Pah1 in a different way than the Nem1-Spo7 phosphatase complex and it does not function as a phosphatase of Pah1.

### Are there additional *L. pneumophila* effectors which affect PA and DAG levels in host cells?

**Figure 8:**
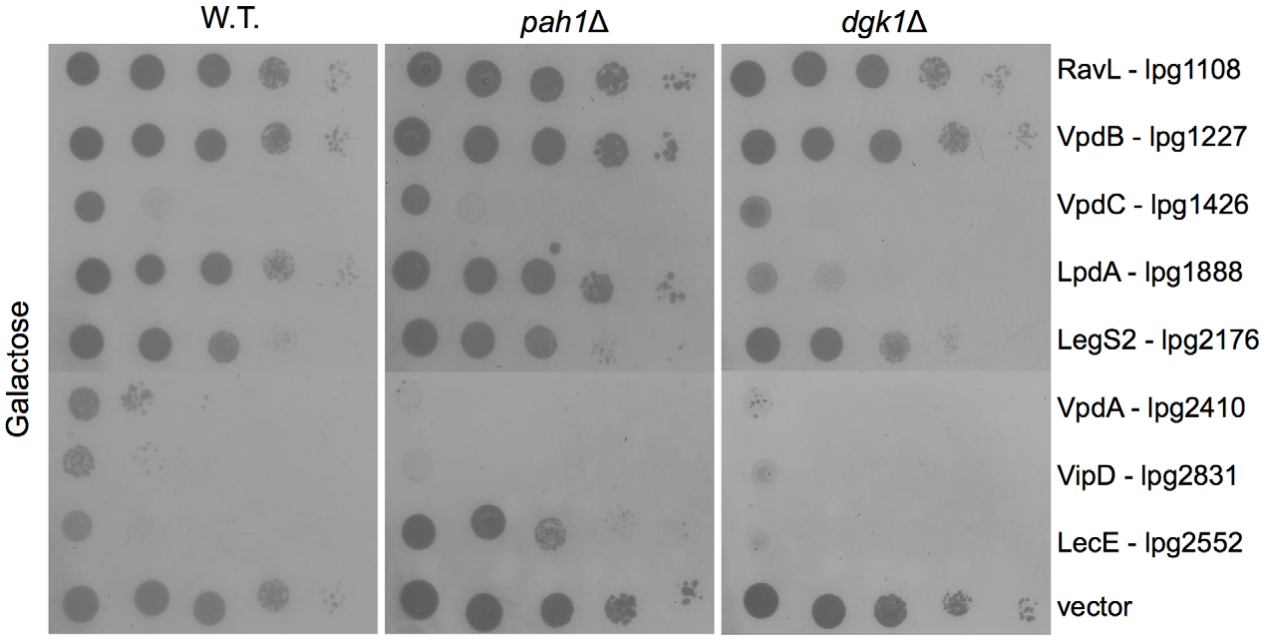
Examination of *L. pneumophila* effectors related to phospholipids biosynthesis for their lethal effect on yeast growth in the wild-type yeast and in the *pah*1 and *dgk*1 yeast deletion mutants. *L. pneumophila* effectors (indicated on the right) expected to be involved in or that were shown to function in phospholipids metabolism were over expressed under the GAL1 promoter in wild-type *S. cerevisiae* BY4741 (W.T.), the *pah*1 deletion mutant RV-L8-59 (*pah1*Δ) or in the *dgk*1 deletion mutant (*dgk1*Δ). LecE was used as a positive control. pGREG523 (vector) was used as a negative control. The glucose control plates are presented in Fig. S3.

It was shown recently that sometimes several *L. pneumophila* effectors affect the same host cell processes during infection (see Introduction). To determine if there are additional effectors that affect PA and DAG levels, we examined seven additional effectors (Table 2) that according to their sequence homology and/or sequence motifs are expected to be involved with or were shown to function in phospholipids biosynthesis [44], [46]. We reasoned that yeast deletion mutants in specific host factors (such as *dgk*1 and *pah*1) can be used in order to uncover additional effectors that target the same cellular process (for example, other effectors that caused lethal effect on yeast growth might be suppressed by the same yeast strains). Moreover, effectors that originally show no lethal effect on wild-type yeast might cause lethal effect when they will be over expressed in the relevant yeast deletion mutants. Such a result might reveal effectors that target the same cellular process (both effectors might activate or one of them might activate and the other inhibit the same process), during *L. pneumophila* infection. For this purpose we cloned the seven effectors listed in Table 2 under the control of the galactose-regulated promoter (GAL1 promoter) and expressed them in wild-type yeast (Fig. 8 and Fig. S3). Three of these effectors (VipD, VpdA and VpdB) caused strong lethal effect on yeast growth and one effector (LegS2) caused a moderate lethal effect on yeast growth when expressed in wild-type yeast and they were not suppressed by the deletions in *dgk*1 or *pah*1. However, interestingly, when lpg1888 (an effector containing a PLD domain, that we named LpdA, see below) was expressed in wild-type yeast no lethal effect was observed, but when it was expressed in the *dgk*1 deletion mutant clear lethal effect was observed, indicating that this specific yeast genetic background exposed the function of the effector.

**Table 2:** *L. pneumophila* effectors predicted to be involved in phospholipids metabolism.

Lpg#	Name	Predicted/validated function	Lethal effect on W.T. yeast	Effect in Δ*dgk*1 or Δ*pah*1 yeast mutants
lpg1108	RavL	Lysophospholipase (LPL)	No	-
lpg1227	VpdB	Phospholipase A2 (PLA)	No	-
lpg1426	VpdC	Phospholipase A2 (PLA)	Strong	-
lpg1888	LpdA	Phospholipase D (PLD)	No	Lethal effect in Δ*dgk*1
lpg2176	LegS2	Sphingosine-1-phosphate lyase (SPL)	Medium	-
lpg2410	VpdA	Phospholipase A2 (PLA)	Strong	-
lpg2552	LecE	Activator of Pah1 (PAP)	Strong	Reduced lethal effect in Δ*pah*1
lpg2831	VipD	Phospholipase A2 (PLA)	Strong	-

Eukaryotic enzymes containing a PLD domain where shown before to convert phosphatidylcholine (PC) to PA and free choline [72], [73], and the yeast Spo14 is a known PLD enzyme (Fig. 4A). Thus the results obtained with LpdA can be explained in the sense that in the absence of Dgk1 there is no enzyme that can phosphorylate DAG back to PA and under these conditions the activity of LpdA was observed.

### LpdA is an effector that functions together with LecE

**Figure 9:**
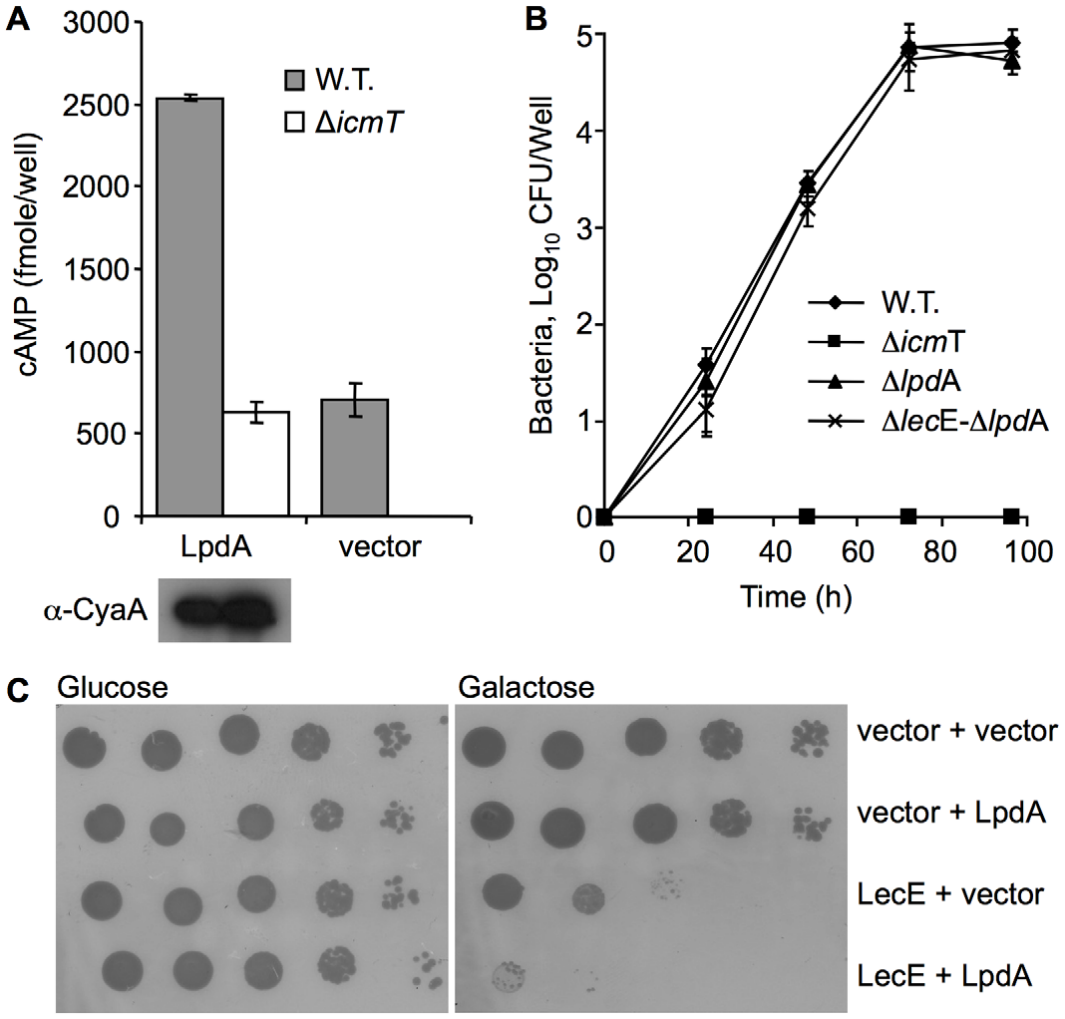
LpdA translocates into host cells and it enhances the LecE lethal effect on yeast growth. (A) LpdA translocates into host cells in an Icm/Dot dependent manner. Wild-type *L. pneumophila* JR32 (gray bars) and *icm*T deletion mutant GS3011 (white bars) harboring the CyaA-LpdA fusion were used to infect HL-60-derived human macrophages and cAMP levels were determined (as described in the Materials and Methods section). The CyaA vector was used as a negative control. The protein levels of the CyaA-LpdA fusion was monitored by western blot analysis using α-CyaA antibody and are presented below each bar. (B) Deletion of *lpd*A from *L. pneumophila* genome or the double *lpd*A/*lec*E deletion mutant causes no intracellular growth defect in the amoebae *A. castellani*. The wild-type *L. pneumophila* JR32 (diamonds), the *lpd*A deletion mutant RV-L10-71 (triangles), the *lpd*A/*lec*E double deletion mutant RV-L10-77 (exes) and the *icm*T deletion mutant (squares) were examined. (C) LpdA enhances the lethal effect on yeast growth caused by LecE. LecE, LpdA or both effectors together were over expressed in wild-type *S. cerevisiae* BY4741. pGREG523 (vector) and pGREG536 (vector) were used as negative controls of LecE and LpdA, respectively.

LpdA was shown before to translocate into host cell, as part of a large screen, and its translocation level was very low (only 5% of the cells show indication for translocation) [19]. Therefore, we fused LpdA to the CyaA reporter and examined its translocation into host cell (Fig. 9A). Our analysis confirms that LpdA translocates into host cells, its translocation levels were low in comparison to the other effectors examined in this study (Fig. 1A), but no translocation was observed from an Icm/Dot deletion mutant (Fig. 9A). To investigate the relations between LecE and LpdA we constructed a single deletion mutant in *lpd*A as well as a double deletion mutant of *lec*E and *lpd*A and examined the intracellular multiplication of these mutants in *A. castellanii*. As can be seen in Fig. 9B, no intracellular growth phenotype was observed for the single or double deletion mutants, as was shown before for most of the deletion mutants in *L. pneumophila* effectors.

To further explore the relations between LpdA and LecE we expressed both proteins together in yeast. This analysis resulted with an additive effect on yeast growth, both effectors together were more lethal to yeast in comparison to LecE by itself (Fig. 9C), indicating that both effectors function in the same direction (LpdA by itself caused no yeast growth defect (Fig. 9C)). Our results reveal two conditions under which LpdA lethal effect on yeast growth can be observed: i) in a *dgk*1 deletion mutant (Fig. 8) and ii) when LecE was expressed in the yeast cells (Fig. 9C). Importantly, both these conditions have the same outcome on the yeast cell since in the first condition the yeast cell cannot convert DAG into PA and therefore DAG probably accumulates in the yeast cell. In the second condition there is high activity of Pah1 due to the expression of LecE that also leads to the accumulation of DAG. Thus, the results obtained with LpdA further supports the function of LecE as a Pah1 activator.

### LpdA possesses a phospholipase-D (PLD) activity

**Figure 10:**
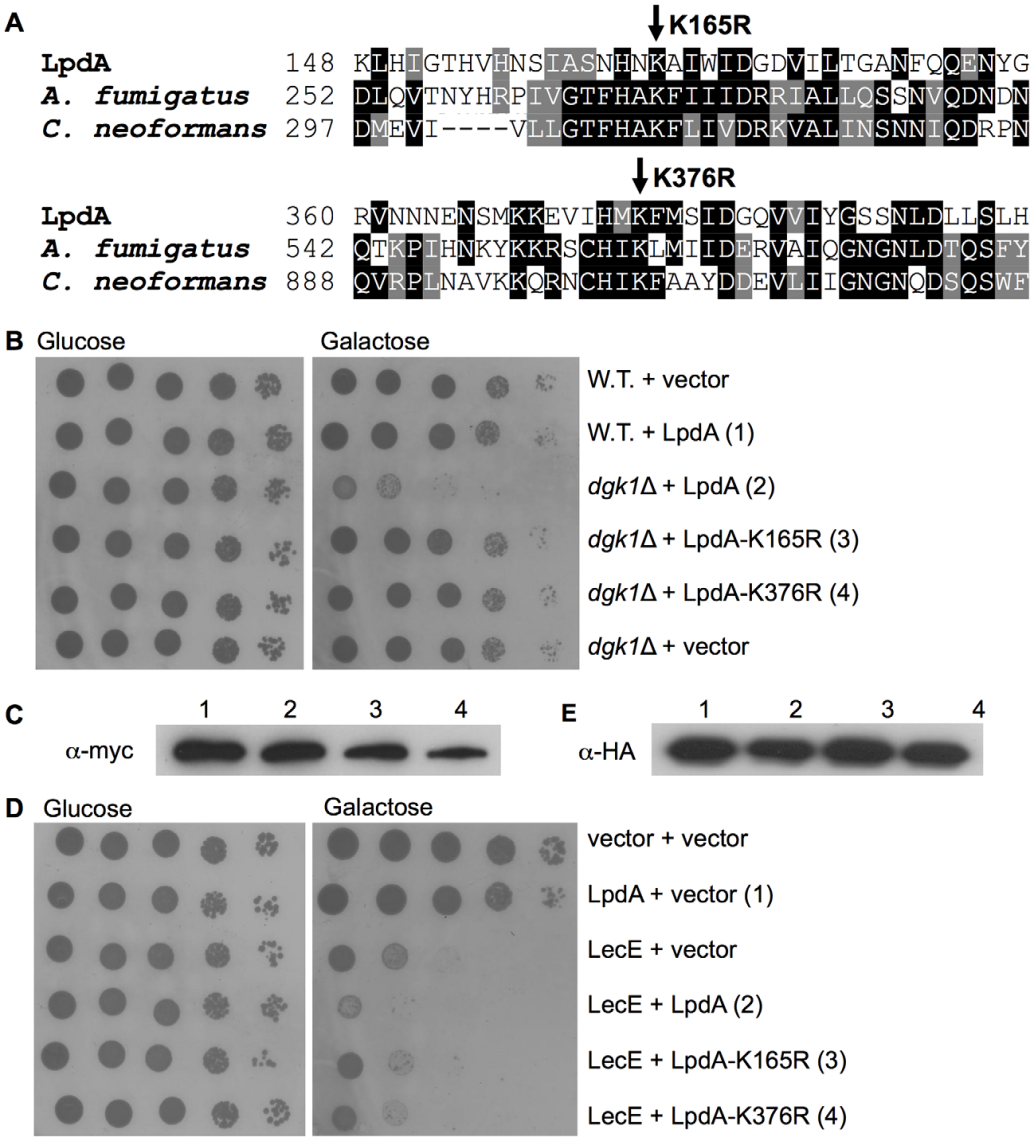
The phospholipase D activity of LpdA is required for its function. (A) Part of a protein sequence alignment of the domains harboring the two HKD motifs of LpdA and its closest homologs from the fungi *Aspergillus fumigatus* and *Cryptococcus neoformans*. The amino-acids K165 and K376 that were mutated are marked by arrows. (B) Point mutations in the suspected PLD active site of LpdA abolished its lethal effect in yeast *dgk*1 deletion mutant. LpdA or its point mutants LpdA-K165R and LpdA-K376R were over expressed in a wild-type *S. cerevisiae* BY4741 (W.T.) or in the *dgk*1 deletion mutant (*dgk1*Δ). K165R and K376R are point mutations in the suspected PLD active site of LpdA, which were shown before to be required for the enzymatic activity of PLD enzymes [74]. pGREG523 (vector) was used as a negative control. (C) The *myc*-tagged LpdA protein levels were monitored by western blot analysis using α-*myc* antibody. The numbers correspond to these in section B. (D) The LpdA PLD activity is required for its enhancement of the LecE lethal effect on yeast growth. LecE was over expressed in wild-type *S. cerevisiae* together with LpdA or with its point mutants LpdA-K165R and LpdA-K376R. pGREG523 (vector) and pGREG536 (vector) were used as negative controls of LecE and LpdA, respectively. (E) The HA-tagged LpdA protein levels were monitored by western blot analysis using α-HA antibody. The numbers correspond to these in section D.

LpdA was suggested to encode for a phospholipase-D due to sequence homology to eukaryotic (fungal) PLD enzymes. The PLD protein family is conserved from yeast to human and it comprises a conserved catalytic core (HxK(x)_4_D) [74]. To determine if LpdA encodes a functional PLD enzyme we generated two point mutations (K165R and K376R) in two conserved lysine residues located in both predicted PLD conserved catalytic cores (Fig. 10A). We then used the LpdA lethal effect observed in the yeast *dgk*1 deletion mutant (Fig. 8) in order to examine these two mutants. As can be seen in Fig. 10B, over expression of the wild-type LpdA in the *dgk*1 deletion mutant caused lethal effect on yeast growth and this effect disappeared when the two LpdA mutants were used, and the yeast growth with these two mutants was similar to the one of the empty vector. These mutations did not detectably affect the stability of LpdA in yeast (Fig. 10C), suggesting that the loss of toxicity was very likely due to the abolishment of the enzymatic activity of LpdA. Due to these results Lpg1888 was named LpdA for *Legionella* Phospholipase D.

As indicated above, LpdA enhances the lethal effect caused by LecE on yeast cells (Fig. 9C). To determine if this enhancement also requires the PLD activity of LpdA, LecE was expressed together with LpdA and it's two mutants (K165R and K376R) in yeast cells. As can be seen in Fig. 10D, the enhancement of the LecE lethal effect by LpdA requires its PLD activity, and the mutations in the PLD active site almost eliminated the enhancement of the lethal effect caused by LpdA. Also in this analysis the two mutations did not detectably affect the stability of LpdA (Fig. 10E).

### LecE influences the localization and accumulation of a DAG sensor *in-vivo*

**Figure 11:**
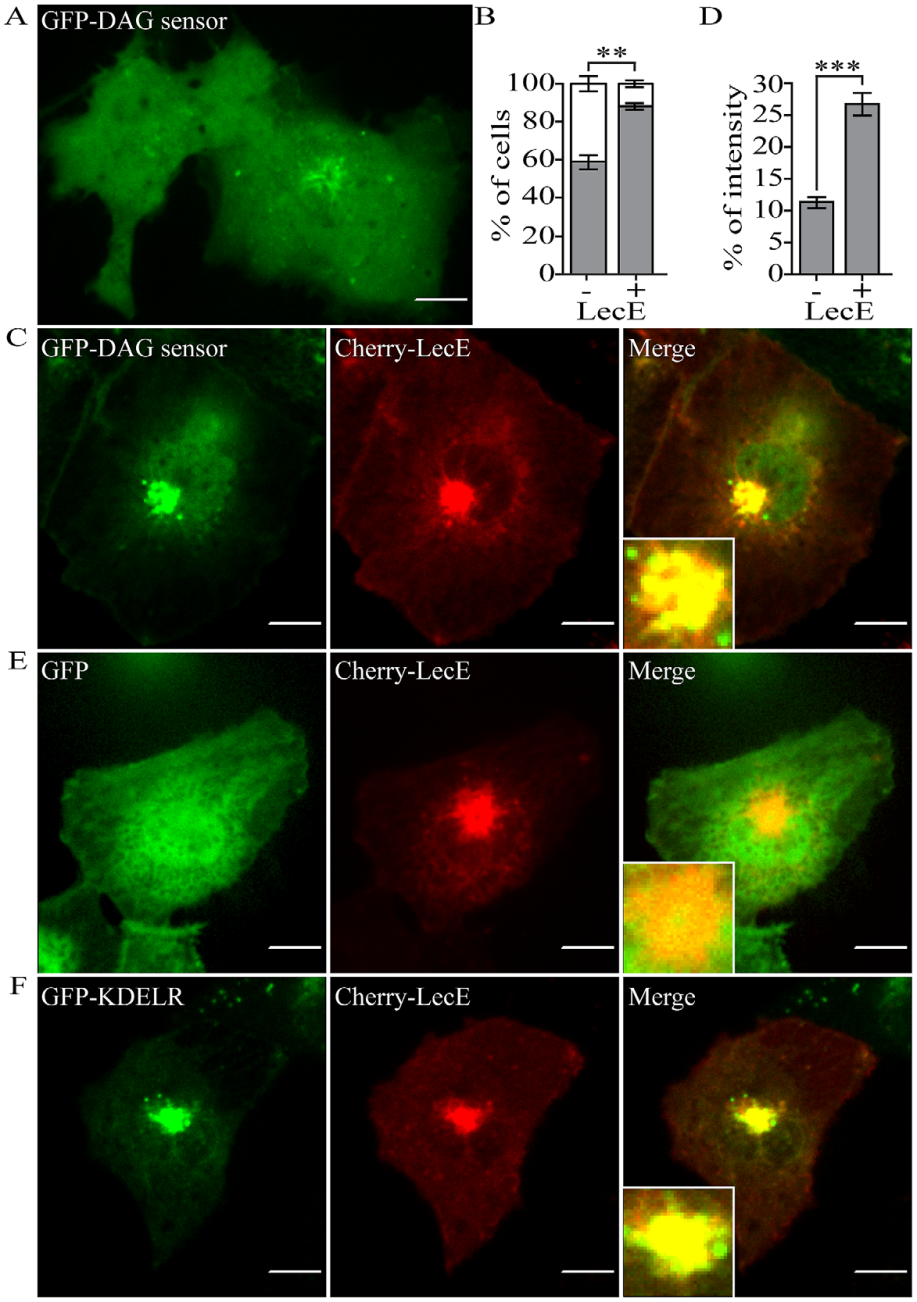
LecE alters the distribution of a GFP-DAG sensor in COS7 cells. When expressed alone the GFP-DAG sensor (green) showed two localization patterns in COS7 cells (A, B), a diffuse pattern (left cell in panel A and white bar in panel B) and a pattern in which it was diffuse in the cell but was also concentrated in a membranal peripheral nucleus area (right cell in panel A and grey bar in panel B). These two patterns were also observed when the GFP-DAG sensor was expressed together with Cherry-LecE (red) (C). The percentage of cells (% of cells) that showed each pattern of the GFP-DAG sensor was determined in cells that expressed the GFP-DAG sensor by itself (−) or with Cherry-LecE (+). Three independent experiments were preformed and the number of cells which showed each of these patterns with and without Cherry-LecE was found to be significantly different (** - *p*<0.007, Student's *t*-test). (D) Quantification of the GFP-DAG sensor accumulation at the peripheral nucleus area of COS7 cells, presented as the ratio between the green fluorescence intensity in the peripheral nucleus area to the total cell intensity of the GFP signal (% of intensity), was preformed in cells that expressed the GFP-DAG sensor by itself (−) or with Cherry-LecE (+). The difference between cells with or without Cherry-LecE was found to be significantly different (2.35 fold, *** - *p*<1.2×10^−10^, Student's *t*-test). Cherry-LecE was also expressed in COS7 cells together with GFP as a negative control (E) or with the cis-Golgi marker GFP-KDEL-Receptor (GFP-KDELR) (F) to determine its localization. The images were acquired using confocal microscopy as described in the Materials and Methods section. The scale bar = 10 µm.

The results obtained from the yeast analysis of LecE indicated that the function of this effector probably results in an increase in DAG levels in cells (Fig. 4). To determine if LecE affects DAG levels *in-vivo* in mammalian cells a system based on a DAG fluorescence biosensor was employed using live-cell imaging. The LecE effector was fused to the mCherry fluorescent protein (Cherry-LecE) and was ectopically expressed in COS7 cells together with a PKC-C1-DAG binding domain fused to GFP (GFP-DAG) that was validated before as a specific DAG sensor in several systems [75]–[78]. When the GFP-DAG sensor was expressed in COS7 cells it exhibited two localization patterns: in 59% of the cells the sensor was diffusely distributed throughout the cell but was also concentrated in a membranal peripheral nucleus area, while in 41% of the cells the GFP-DAG sensor showed a completely diffuse distribution (Fig. 11A, B). In contrast, when the GFP-DAG sensor was expressed together with Cherry-LecE its distribution changed and in 88% of the cells the GFP-DAG sensor was mostly concentrated in the membranal peripheral nucleus area (Fig. 11B). Moreover, a similar intracellular distribution was also obtained for Cherry-LecE (Fig. 11C). Importantly, the Cherry-LecE induced changes of the GFP-DAG sensor was significant (*p*<value 0.007, Student's *t*-test; Fig. 11B). In addition, the effect of Cherry-LecE on the accumulation of the GFP-DAG sensor in the peripheral nucleus area was examined. In this analysis, the GFP-DAG sensor concentration at the peripheral nucleus area was significantly enriched in cells expressing Cherry-LecE in comparison to cells expressing the GFP-DAG sensor by itself, (2.35 fold, *p*<value 1.2×10^−10^ in Student's *t*-test; Fig. 11D). As a control, GFP was expressed in the presence or absence of Cherry-LecE, and no alterations to the mixed cytosolic and nuclear distribution of GFP were observed upon Cherry-LecE co-expression (Fig. 11E and data not shown). This result further supports the conclusion that Cherry-LecE influences the distribution of the GFP-DAG sensor specifically. In addition, the specific localization of Cherry-LecE was examined and it was found to be localized to the cis-Golgi apparatus as it co-localized with GFP-KDEL-Receptor (GFP-KDELR) a well established cis-Golgi marker (Fig. 11F) [79]. Notably, a previous work done with a different GFP-DAG sensor (based on the PKD-C1-DAG binding domain) found it to be localized to the Golgi in HeLa cells [80]. The combined results presented demonstrate that LecE induces alternations in DAG content in COS7 cells and show co-localization of the ectopically expressed effector and its lipid product to the same sub-cellular compartment, the cis-Golgi.

### LpdA influences the localization of a PA sensor *in-vivo*

**Figure 12:**
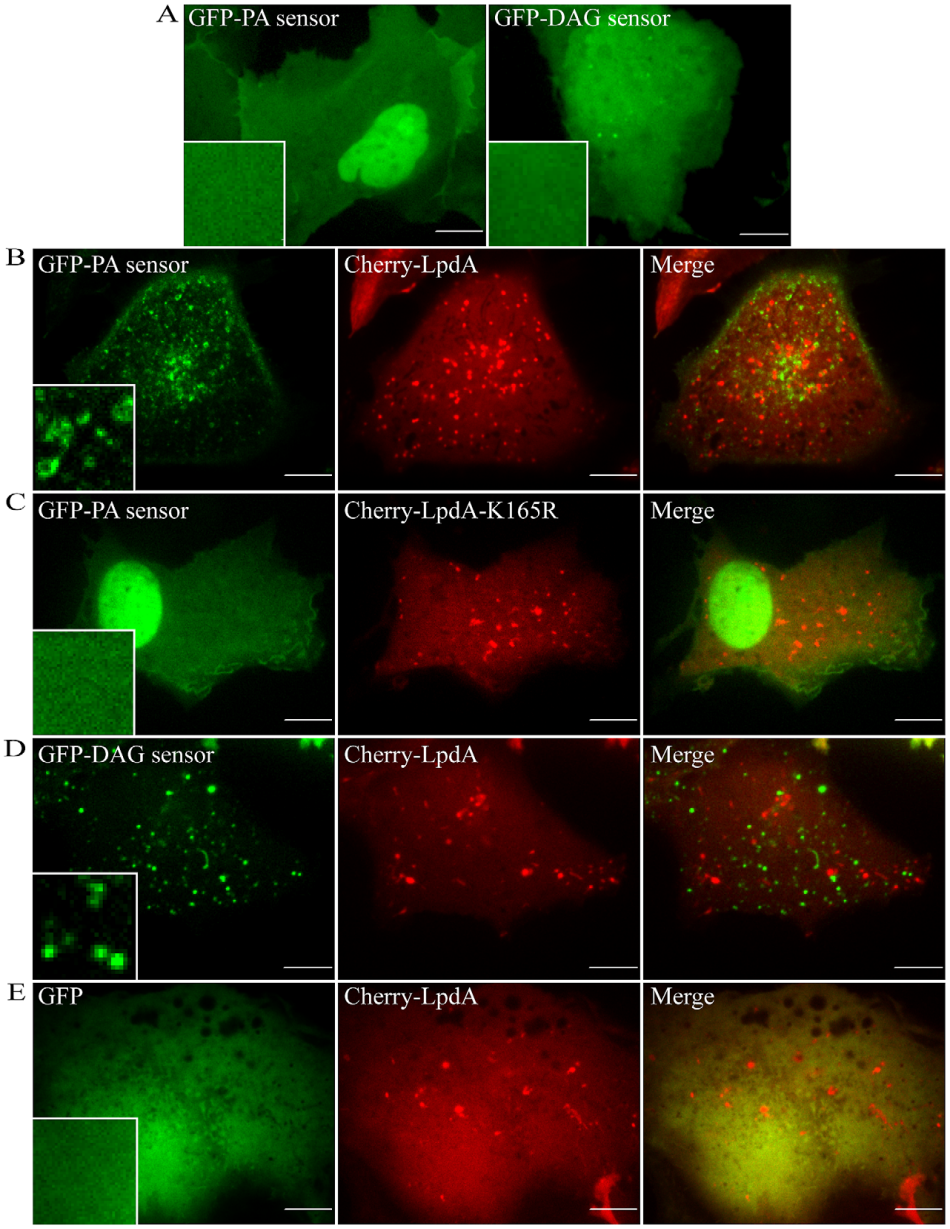
LpdA influences the localization of a PA sensor and a DAG sensor in COS7 cells. The GFP-PA sensor (green) or the GFP-DAG sensor (green) were ectopically expressed in COS7 cells by themselves (GFP-PA sensor on the left and GFP-DAG sensor on the right, of panel A) or together with Cherry-LpdA (red) (B, D). The GFP-PA sensor was also expressed with the LpdA PLD mutant, Cherry-LpdA-K165R (C), and the Cherry-LpdA was also expressed with GFP (E). Cherry-LpdA showed clear change in the localization of both the GFP-PA and GFP-DAG sensors while no alterations of the GFP distribution were observed upon co-expression. The images were acquired using confocal microscopy as described in the Materials and Methods section. The scale bar = 10 µm.

An analogous approach to the one described above was also applied to address the functionality of LpdA in mammalian cells and its ability to influence the levels and distribution of PA. For that purpose, LpdA was fused to the mCherry fluorescent protein (Cherry-LpdA) and ectopically expressed in COS7 cells together with GFP fused to a PA-binding domain from the yeast Spo20 SNARE protein (GFP-PA). This domain was previously shown to function as a sensitive and specific PA sensor in mammalian cells [81]. As shown in Fig. 12A (on the left), when the PA sensor was expressed by itself it accumulated in the cell nucleus. Several studies have shown before a similar accumulation of the GFP-PA sensor in resting cells, and it was found to be not specific [81], [82]. In striking contrast, when Cherry-LpdA was expressed together with GFP-PA sensor it induced a punctuate distribution of GFP-PA throughout the cell's cytoplasm (Fig. 12B), suggesting an effector-dependent generation of PA. Of note, the GFP-PA-labeled structures were highly mobile, resembling intracellular vesicles. The Cherry-LpdA effector itself showed a diffuse pattern in the cells with some punctuate distribution as well (Fig. 12B). Importantly, when the GFP-PA sensor was expressed together with the LpdA PLD mutant, Cherry-LpdA-K165R, no change in the distribution of the GFP-PA sensor was observed (Fig. 12C), what indicates that the PA production in the cells depended on the PLD activity of the Cherry-LpdA. In addition, it was demonstrated before that PA is usually dephosphorylated to DAG *in-vivo* [83], [84]; thus, we examined the distribution of the GFP-DAG sensor (described in the previous section) when co-expressed with Cherry-LpdA, and found that it was also localized to motile puncta (Fig. 12D), in sharp contrast to its distribution pattern when expressed alone (Fig. 11A and Fig. 12A on the left). Importantly, when GFP was expressed with or without Cherry-LpdA it showed a diffuse distribution in the cells with some concentration in the cell nucleus (Fig. 12E and data not shown). The combined results presented indicate that the changes observed with LpdA were specific to the PA and DAG sensors. These results substantiate LpdA as a PLD enzyme in the cells, where it generates PA which is further converted to DAG.

### LecE and LpdA are localized to the LCV during infection

**Figure 13:**
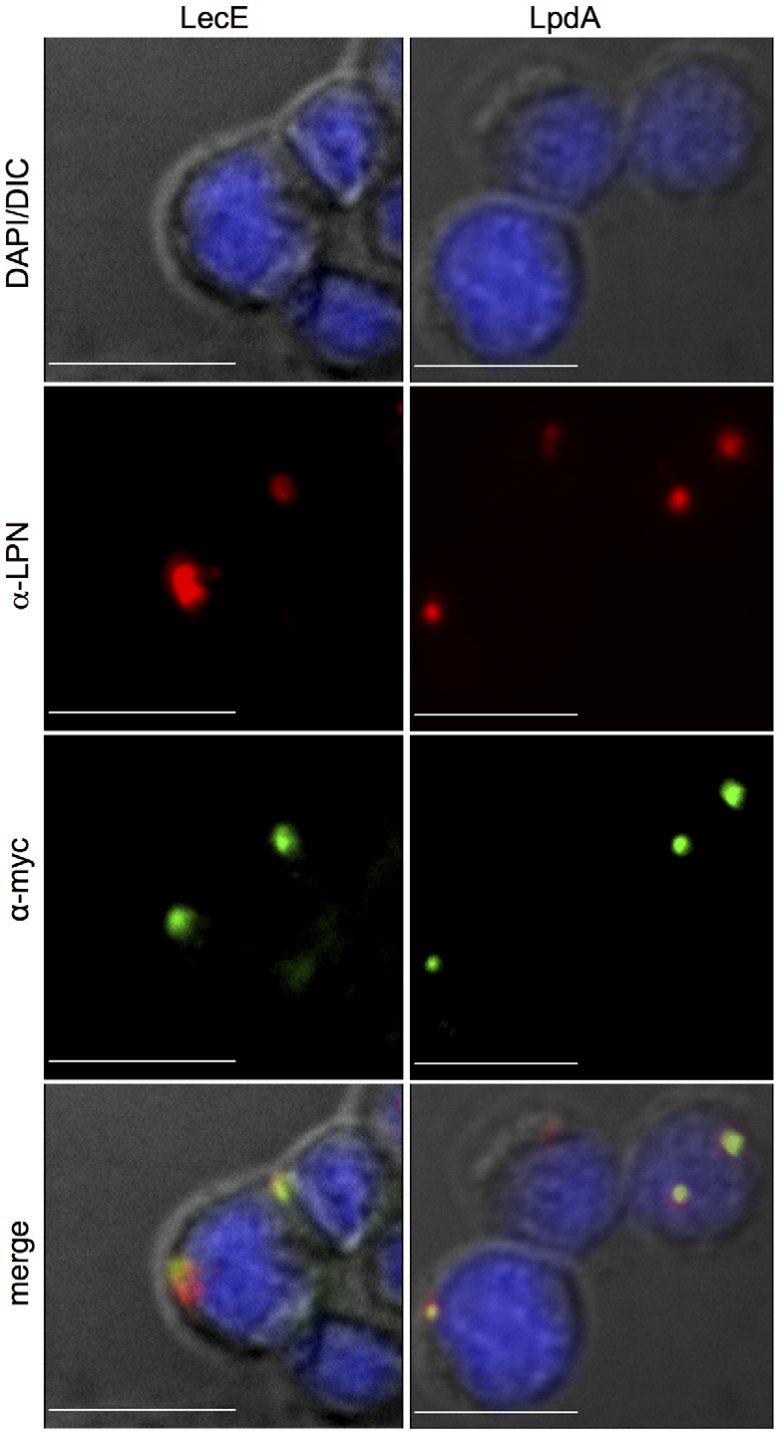
LecE and LpdA are both localized to the LCV during infection. U937-derived-human macrophages were infected with wild-type *L. pneumophila* JR32 expressing 13×*myc* tagged LecE or LpdA. Cells were then stained with α-*myc* and Alexa-488 antibodies (green), with α-*L. pneumophila* (α-LPN) and Cy3 antibodies (red) and with DAPI (blue). Representative images show clear colocalization (yellow) of both LecE and LpdA with the bacterial phagosome (merge-right panel). The images were acquired using confocal microscopy as described in the Materials and Methods section. The scale bar = 10 µm.

To determine where in the host cell LecE and LpdA perform their function during *L. pneumophila* infection, we constructed plasmids that over express these effectors in *L. pneumophila* as a fusion to a myc-tag at their N-terminus, and infected U937-derived human macrophages with a wild-type *L. pneumophila* containing these plasmids and used confocal fluorescence microscopy to visualize the two effectors during infection. As can be seen in Fig. 13, both effectors were found to be localized to the LCV during infection. Only intracellular bacteria show a signal with the anti-myc antibody directed against the effectors.

Thus we conclude that LecE and LpdA are both localized to the LCV, where they probably manipulate the phagosome phospholipids composition during infection.

## Discussion

**Figure 14:**
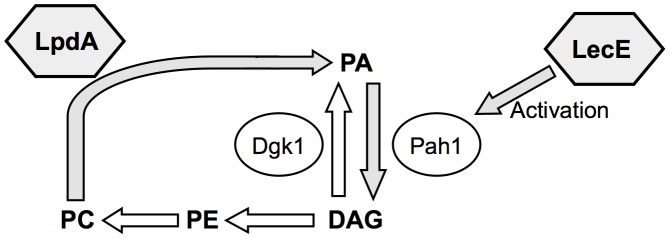
Two *L. pneumophila* effectors influence the PA-DAG biosynthetic pathway in yeast. The effectors LecE and LpdA are indicated by grey hexagons near the steps in the lipids biosynthesis pathway they affect (grey arrows). Lipid compounds are marked in uppercase letters and bold case. Enzymes known to catalyze individual steps in the phospholipids biosynthesis pathways in yeast are indicated in circles. PA, phosphatidic acid; DAG, diacylglycerol; PE, phosphatidylethanolamine; PC, phosphatidylcholine. Dgk1 is a DAG-kinase and Pah1 is a PA-phosphatase.

Up to date about 300 effector proteins were identified in *L. pneumophila* and the function of only several of them was uncovered. Effectors were found to affect diverse host cell processes which include vesicular trafficking, apoptosis, ubiquitination, translation and others [7], [85]. In several cases, pairs of effectors were found to function together and one effector was found to counteract the function of another effector. The SidM/DrrA effector was found to AMPylate the host cell small GTPase binding protein Rab1, thus keeping it in an active state which cannot be inactivated by host cell factors [23], and the effector SidD was found to reverse this modification by deAMPylation of Rab1 [24], [86]. Another pair of effectors also involved in Rab1 activation was described recently - AnkX and Lem3. AnkX was found to phosphocholinate Rab1 and Lem3 was found to reverse this modification [25]–[27]. An additional effector that was found to affect another effector is LubX. LubX contains an E3 ubiquitin ligase domain and it was found to specifically target the bacterial effector protein SidH for degradation by the host cell proteasome [31], thus affecting the time during infection when SidH is present in the host cell and performs its function. In this manuscript, we described a new pair of effectors that might function together – LecE and LpdA. This pair of effectors is different from the three pairs described above since both effectors function in the same direction and do not counteract the function of one another. The effector protein LpdA was found to contain a functional PLD domain and these enzymes were shown before to convert PC to PA and free choline [87]. The second effector – LecE was found to activate the yeast lipin homolog (Pah1) which converts PA to DAG (Fig. 14). Both these effectors were found to be localized to the LCV during infection thus the combined lipid biosynthetic reactions that might occur on the LCV will include conversion of PC into PA (by LpdA) and then conversion of PA to DAG (by LecE activated PA phosphatase) a process which is expected to result in changes of the lipid composition of the LCV that can affect its fate in the host cell as well as the host proteins and bacterial effectors that will be recruited to the LCV (see below). These results indicate that pairs or groups of *L. pneumophila* effectors function together and additional such effectors are expected to be found.

The way by which LecE activates Pah1 is currently not known. The natural activation of Pah1 in yeast occurs via dephosphorylation, but our results indicate that this is not the way by which LecE activates Pah1 (Fig. S2). An important result regarding the mode of activation by LecE comes from the finding the over-expression of Pho80-Pho85 in yeast suppresses the lethal effect caused by LecE. This result suggests that the activity of Pho80-Pho85 is dominant on the activity of LecE, therefore it might be that LecE cannot perform its function when Pah1 is fully phosphorylated (the expected state of Pah1 after Pho80-Pho85 over-expression). We hypothesize that LecE activates Pah1 by modifying one of its amino acids (as was shown for SidM and AnkX in the case of Rab1) or by directly binding to it.

Identification of pairs or groups of effectors that influence the same or related host cell processes is very important for the ability to understand the function of the enormous number of effectors translocated by *L. pneumophila* during infection. The approach that we used in this study, which led to the identification of LpdA as an effector that function with LecE, can help to discover such pairs and/or groups of effectors. Our approach takes advantage of yeast genetics as a tool to identify such groups of effectors. This approach can be applied in a very broad way in order to study effector proteins. When an effector that causes lethal effect on yeast growth is found and a yeast suppressor is identified, other effectors that cause yeast lethal effect and might be suppressed by the same yeast suppressor can be identified in case that they affect the same host factor in a similar way (activation or inactivation). For example the lethal effect on yeast growth caused by AnkX was found to be completely suppressed by over expression of the yeast Ypt1 protein (the yeast homolog of Rab1) [27], it is possible that other effectors that cause lethal effect on yeast growth will be suppressed by over expression of Ypt1, thus leading to the identification of the cellular process they affect. An even more interesting situation is the one described in this manuscript. LpdA causes no lethal effect on wild-type yeast, but when it was expressed in a yeast *dgk*1 deletion mutant clear lethal effect was observed. In this way, not only effectors that cause lethal effect on wild-type yeast can be sorted into functional groups but also effectors that cause no lethal effect on yeast growth can be sorted, since their effect can be uncovered by using different yeast genetic backgrounds. In the case of LecE and LpdA, over expression of Dgk1 suppresses the lethal effect of LecE and a deletion of *dgk*1 uncovered the lethal effect of LpdA thus indicating that both effectors function in the same direction. Our approach can also be expanded to other host cell processes expected to be affected by *L. pneumophila* effectors (or any other pathogens). For example, yeast deletion mutants or strains over expressing genes related to trafficking (such as *vps*) or authophagy (such as *apg*) can be used to screen the collection of *L. pneumophila* effectors, both the ones that cause lethal effect on wild-type yeast as well as these that have no effect on wild-type yeast growth. In this way pairs or groups of effectors that affect similar host cell processes can be uncovered.

The results presented in this study uncover another aspect of the involvement of phospholipids in *L. pneumophila* infection of host cells. It was shown before that several *L. pneumophila* effectors (SidC, SidM/DrrA and SdcA) specifically bind PI4P on the LCV [47], [48], [88]. However, it is known that PC constitutes the major phospholipid in eukaryotic membranes [89]. The results presented in this study show that the combined activity of the LpdA and LecE effectors is expected to result in the conversion of PC to DAG on the LCV. In addition, it was shown before that the presence of PI4P on the LCV is strongly dependent on the activity of the enzyme PI 4-kinase IIIβ (PI4KIIIβ) that converts PI into PI4P [47]. One way to recruit PI4KIIIβ to the LCV is by the activity of Arf1 [90], however it was shown before that RalF that recruits Arf1 to the LCV is not required for SidC decoration of the LCV [47]. Another, major way to recruit PI4KIIIβ to membranes is by the action of protein kinase-D (PKD). The recruitment of the latter to membranes is mainly mediated by its two DAG C1-binding domains [91]. The activation of PKD also requires phosphorylation by protein kinase-C (PKC) which is also recruited to membranes by DAG [92]. Thus, one way to increase the levels of PI4P on the LCV is by generating higher levels of DAG by the function of LpdA and LecE. The higher levels of DAG will result in the recruitment of PKC and PKD to the LCV, then PKC may phosphorylate PKD that will lead to the recruitment of PI4KIIIβ to the LCV that in turn will generate PI4P from PI. It is important to note that this is probably not the only way by which the LCV can recruit PI4KIIIβ since this enzyme can also be recruited from Golgi derived vesicles that fuse with the LCV.

The results presented in this study uncovered an additional layer in the complex interaction between the *L. pneumophila* phagosome and the host cell, and show that changes in phospholipids composition are manipulated by *L. pneumophila* effectors in many ways to result with successful infection.

## Materials and Methods

### Bacterial and yeast strains, plasmids and primers

The *L. pneumophila* wild-type strain used in this work was JR32 [93], a streptomycin-resistant, restriction-negative mutant of *L. pneumophila* Philadelphia-1, which is a wild-type strain in terms of intracellular growth. In addition, mutant strains derived from JR32, which contain a kanamycin (Km) cassette instead of the *icm*T gene (GS3011) [11], the lpg2552 gene (RV-L6-45) (this study), a gentamicin (Gm) cassette instead of the lpg1888 gene (RV-L10-71) (this study), and a double lpg2552/lpg1888 deletion (RV-L10-77) (this study) were used. The *E. coli* strains used were MC1022 [94] and DH5α. The *S. cerevisiae* wild-type strains used in this work were BY4741 (*MAT*a *his3*Δ *leu2*Δ *met15*Δ *ura3*Δ) [95] and W303 (*MAT*a *leu2-3,112 trp1-1 can1-100 ura3-1 ade2-1 his3-11,15*) [96]. In addition, mutant strains derived from BY4741, which contain a G418 cassette instead of the *pah*1 gene (RV-L8-59) (this study), the *dgk*1 gene [97] (a kind gift from Prof. Martin Kupiec, Tel-Aviv University) and the *nem*1 gene (RV-L8-54) (this study) were used. Plasmids and primers used in this work are listed in Table S1 and S2.

### Construction of CyaA and 13× myc fusions

The pMMB-cyaA-C vector [98] was used to construct CyaA fusions. In addition, two plasmids were constructed to contain the pUC-18 polylinker, at the same reading frame like pMMB-cyaA-C, in order to generate C-terminal fusions. For the over expression of effectors in yeast the pUC-18 polylinker was cloned into pGREG523 [99], between the EcoRI and HincII restriction sites to generate pRam (this vector was used to construct 13× *myc* fusions under the yeast GAL1 promoter). For the effectors localization experiments the 13× *myc* tag was amplified by PCR from pRam using the primers Myc-F-NdeI and Myc-R-yeast, and the PCR product was digested with NdeI and EcoRI. The pUC-18 polylinker was digested from pMMB-cyaA-C with EcoRI and BamHI and the resulting inserts were cloned in a 3-way ligation into pMMB207-NdeI [98], digested with NdeI and BamHI, to generate pMMB-13× *myc* (this vector was used to construct 13× *myc* fusions under the bacterial P*tac* promoter).

The *L. pneumophila* genes examined were amplified by PCR using a pair of primers containing suitable restriction sites (Table S2). The PCR products were subsequently digested with the relevant enzymes, and cloned into pUC-18. The plasmids inserts were sequenced to verify that no mutations were introduced during the PCR. The genes were then digested with the same enzymes and cloned into the suitable plasmids described above. Lpg1888 was also cloned into pGREG536 that contain the same reading frame as the above mentioned vectors to generate pGREG536-1888 (generating a 7xHA fusion under the yeast GAL1 promoter).

### Construction of plasmids expressing *S. cerevisiae* genes

The *pah*1 gene was amplified by PCR with its native promoter using the Pah1-for and Pah1-rev primers. The PCR product was cloned into pUC-18, sequenced, and then digested out from pUC-18 using XbaI and PvuII. C-terminal 3xHA tag was amplified by PCR with the primers HA-for and HA-rev using the pYM1 plasmid [100] as template, followed by cloning into pUC-18, sequencing and digest with XbaI and SalI. Both inserts were then cloned into pGREG505 digested with Ecl136 and SalI, in a 3-way ligation, to generate pGREG505-Pah1-3xHA. The genes *dgk*1, *spo*7, *nem*1, *pho*80 and *pho*85 were amplified by PCR using the DGK1-SpeI and DGK1-SalI primers for *dgk*1, the SPO7-SpeI and SPO7-SalI primers for *spo*7, the Nem1-SpeI and Nem1-SalI primers for *nem*1, the Pho80-SpeI and Pho80-SalI primers for *pho*80 and the Pho85-SalI-for and Pho85-SalI-rev primers for *pho*85 (Table S2). The PCR products were cloned into pUC-18, sequenced, and then digested out from pUC-18 using SpeI and SalI (for *pho*85 only SalI was used), followed by cloning into different vectors from the pGREG series [99] digested with the same enzymes; pGREG506 for *dgk*1 to generate pGREG506-Dgk1, pGREG505 or pGREG506 for *nem*1 to generate pGREG505-Nem1 and pGREG506-Nem1, respectively, pGREG503 or pGREG505 for *spo*7 to generate pGREG503-Spo7 and pGREG505-Spo7, respectively, pGREG504 or GREG505 for *pho*80 to generate pGREG504-Pho80 and pGREG505-Pho80, respectively and pGREG506 for *pho*85 to generate pGREG506-Pho85.

### Construction of mCherry fusions

The plasmid pmCherryC1-hMPV [101], that contains an EcoRI site at the same reading frame like in pUC-18, was used in order to construct C-terminal mCherry fusions under the viral pCMV promoter, using the same restriction enzymes as was mentioned above for both lpg2552 and lpg1888, to generate the plasmids listed in the Table S1.

### Cloning for *L. pneumophila* allelic exchange

Fragments of 1 kb from the upstream and the downstream regions of the lpg2552 and lpg1888 genes were amplified by PCR using genomic *L. pneumophila* DNA as a template and pairs of primers containing suitable restriction sites (Table S2). The resulting fragments were digested with the appropriate enzymes and cloned into pUC-18 to generate pRV-lpg2552-UP and pRV-lpg2552-DW, respectively, for lpg2552, and pRV-lpg1888-UP and pRV-lpg1888-DW, respectively, for lpg1888, and sequenced. These two pairs of plasmids were then digested with the respective restriction enzymes and cloned into pUC-18 together with the Km resistance cassette digested with SalI to generate pRV-lpg2552-KM, for lpg2552, or together with the Gm resistance cassette digested with EcoRV to generate pRV-lpg1888-GM, for lpg1888. The two fragments containing the upstream region, the downstream region and the Km/Gm cassette between them were digested with PvuII or SmaI, respectively, and cloned into pLAW344 digested with EcoRV to generate pRV-lpg2552::KM-del and pRV-lpg1888::GM-del, for lpg2552 and lpg1888, respectively. These two plasmids were used for allelic exchange as previously described [102]. For the construction of the double lpg2552::Km/lpg1888::Gm deletion mutant, pRV-lpg1888::GM-del was used to generate the lpg1888::Gm deletion in the lpg2552::Km deletion mutant (RV-L6-45). These strains were examined for intracellular growth in *A. castellanii* as previously described [103].

### Construction of *S. cerevisiae* deletion mutants

In order to construct yeast deletion mutants in the genes *pah*1 and *nem*1, a KanMX resistance cassette was amplified by PCR from pM4754 [104] using primer containing the first and last 50 bp of each gene; Pah1-kanMX-for and Pah1-kanMX-rev for *pah*1, and NEM1-kanMX-for and NEM1-kanMX-rev for *nem*1. The PCR products were then ethanol precipitated, transformed into wild-type yeast using standard lithium acetate protocol [105], spotted on YPD plates (20 gr glucose, 10 gr yeast-extract, 20 gr peptone in 1 L of distilled H_2_O) that contained 200 µg/ml G418 and incubated for 2–3 days at 30°C, followed by replica plating on similar plates and incubation for additional 2–3 days at 30°C. Single colonies were then isolated on similar plates and the deletions were verified by PCR.

### Site directed mutagenesis

In order to mutate specific amino acids in the active sites of lpg1888, Pah1 and Dgk1 the PCR overlap-extension approach was used [106], in a similar way as described before [98]. For the construction of site specific mutants in the putative PLD active sites of lpg1888, the primers lpg1888-K165R-F and lpg1888-K165R-R were used to generate 1888-K165R and the primers lpg1888-K376R-F and lpg1888-K376R-R were used to generate 1888-K376R. For the construction of site specific mutant in the Pap1 active site of Pah1, the primers Pah1-D398E-for and Pah1-D398E-rev were used to generate Pah1-D398E. For the construction of site specific mutants in the diacylglycerol kinase active sites of Dgk1, the primers Dgk-R76A-F and Dgk-R76A-R were used to generate Dgk1-R76A and the primers Dgk-D177A-F and Dgk-D177A-R were used to generate Dgk1-D177A.

### Western blot analysis

For all protein fusions described above, the formation of a fusion protein with a proper size was validated by Western blot analysis using the anti CyaA antibody 3D1 (Santa Cruz Biotechnology, Inc.) in the case of the CyaA fusions, using the anti *myc* antibody 9E10 (Santa Cruz Biotechnology, Inc.) in the case of the 13× *myc* fusions or using the anti HA antibody F-7 (Santa Cruz Biotechnology, Inc.) in the case of the HA tag fusions. In all cases the primary antibody was diluted 1∶500 and goat anti-mouse IgG conjugated to horseradish peroxidase (Jackson Immunoresearch Laboratories, Inc.) diluted 1∶10,000 was used as the secondary antibody.

### CyaA translocation assay

Differentiated HL-60-derived human macrophages plated in 24-wells tissue culture dishes at a concentration of 2.5×10^6^ cells/well were used for the assay. Bacteria were grown on CYE (ACES-buffered charcoal yeast extract) plates containing chloramphenicol for 48 h. The bacteria were scraped off the plates and suspended in AYE (ACES-buffered yeast extract) medium, the optical density at 600 nm (OD600) was adjusted to 0.1 in AYE containing chloramphenicol, and the resulting cultures were grown on a roller drum for 17 to 18 h until an OD600 of about 3 (stationary phase) was reached. The bacteria were then diluted in fresh AYE medium to obtain an OD600 of 0.2 and grown for 2 h. IPTG was added to final concentration of 1 mM, and the cultures were grown for additional 2 h. Cells were infected with bacteria harboring the appropriate plasmids at a multiplicity of infection of 4, and the plates were centrifuged at 180×*g* for 5 min, followed by incubation at 37°C under CO_2_ (5%) for 2 h. Cells were then washed twice with ice-cold PBS (1.4 M NaCl, 27 mM KCl, 100 mM Na_2_HPO_4_, 18 mM KH_2_PO_4_) and lysed with 200 µl of lysis buffer (50 mM HCl, 0.1% Triton X-100) at 4°C for 30 min. Lysed samples were boiled for 5 min and neutralized with NaOH. 110 µl of each sample was then transferred to a new tube and 220 µl of cold 95% ethanol was added. Samples were then centrifuged for 5 min at 4°C and the supernatant was transferred to a new tube and stored at −20°C until the next step was performed. The samples were dried in a speed-vac and suspended in 110 µl of sterile DDW. Samples were incubated at 42°C for 5 min, followed by 5 min incubation at room temperature. The levels of cyclic AMP (cAMP) were determined using the cAMP Biotrak enzyme immunoassay system (Amersham Biosciences) according to the manufacturer's instructions.

### Yeast lethality assay

*L. pneumophila* effectors encoding genes and *S. cerevisiae* encoding genes were cloned under the GAL1 promoter in the pGERG yeast expression vectors series as described above. Plasmids were transformed into yeast cells using standard lithium acetate protocol [105], and transformants were selected for the appropriate prototrophy on minimal SD (synthetic defined) dropout plates (20 gr glucose, 6.7 gr yeast nitrogen base, 20 gr agar, 1.5 gr amino-acids mixture without the selective ones, in 1 L of distilled H_2_O). Resulting transformants were then grown over-night in liquid SD culture medium at 30°C, cell number was adjusted and a series of tenfold dilutions were made. The cultures were then spotted onto the respective SD dropout plates containing 2% glucose or galactose. Plates were incubated at 30°C or 37°C for 2–3 days and visualizes for differences in growth.

### High copy number suppressor screen for lpg2552

Wild-type *S. cerevisiae* expressing lpg2552 from the GAL1 promoter (pRam-lpg2552) was transformed with a Yep24 based, high copy number, yeast genomic library [107]. About 160,000 transformants were screened for their ability to suppress the toxicity of the lpg2552 over expression on galactose plates at 37°C for three days, and 23 suspected colonies were then isolated twice on similar plates. The suspected suppressors were then subjected to Western-blot analysis in order to confirm that lpg2552 is still intact, and only three suppressors gave a positive result, “Sup-1”, “Sup-13” and “Sup-14”. The library plasmid was recovered from each of these suppressor colonies and re-transformed into the original screening strain to verify the suppression effect. Two of these suspects- “Sup-13” and “Sup-14”, kept the suppressor phenotype at this stage. Sequencing of “Sup-14” reveled that the genomic fragment cloned in the plasmid contained the yeast HIS3 gene and therefore it was left out (HIS3 was the marker that was used to keep lpg2552 plasmid in the yeast cells). “Sup-13” was sequenced and found to contain a fragment of the yeast genome and three sub-clones were constructed from it. Digestion of “Sup-13” with PvuII and self ligation generated pSup-13-sub-clone-1. Digestion of “Sup-13” with SacI and self ligation generated pSup-13-sub-clone-2. Digestion of pSup-13-sub-clone-1 with SmaI and BstEII, followed by treatment with Klenow fragment and self ligation generated pSup-13-sub-clone-3.

### Pah1 band migration assay

*S. cerevisiae* containing plasmids expressing lpg2552, Spo7-Nem1 or a vector were grown on a roller drum over-night in the appropriate SD medium at 30°C. The following day the cultures were centrifuged and resuspended in SD medium containing 2% galactose and the cultures were grown on a roller drum for additional 6 h at 37°C. The cells were then harvested and subjected to SDS PAGE (0.8%) followed by Western-blot analysis using the anti HA antibody.

### Fluorescence analysis of PA and DAG biosensors in COS7 cells

COS7 cells were transfected with the FuGENE (Roche) transfection reagent according to the manufacturer's instructions. Briefly, COS7 cells were grown in DMEM (Invitrogen) medium supplemented with 10% FBS. A day prior to transfection the cells were plated in 6-well plates containing 25 mm glass coverslips at a concentration of 3×10^5^ cells per well. The next day the medium was replaced and the cells were transfected using a total of 1–2 µg DNA per well. Following 44–48 h of incubation at 37°C under CO_2_ (5%), the cells were used for live imaging. The intracellular distribution of the GFP-DAG sensor was classified through: (i) the visual inspection of 330 cells co-expressing Cherry-LecE and GFP-DAG and 473 cells expressing GFP-DAG alone, from three independent experiments; and (ii) the measurement of the ratio of peri-nuclear GFP-DAG to the total cell intensity of the GFP signal; in cells in which a peri-nuclear GFP-DAG signal could be identified. For this quantification, the entire cell volume was imaged, images were projected into two dimensions by summing the pixel intensities of each plane, and GFP signals were identified through intensity-based segmentation. Signal intensities were calculated with Slidebook. Two independent experiments, comprising 40 cells per condition, were performed.

### Fluorescence analysis during *L. pneumophila* infection of U937 cells

Infected cells were visualized by confocal microscopy. Coverslips were inserted into a 24-wells tissue culture dishes and incubated for 1 h with 10% Poly-L-Lysine (Sigma) diluted in PBS (1.5 M NaCl, 78 mM Na_2_HPO_4_, 18.5 mM NaH_2_PO_4_·H_2_O), followed by three washes with PBS. U937 cells were then differentiated into human-like macrophages by addition of 10% normal human serum and 10 ng/ml of phorbol 12-myristate 13-acetate (TPA) (Sigma) at concentration of 0.5×10^6^ cells per well, and incubated at 37°C under CO_2_ (5%) for 48 h. Bacteria were grown as described above for the CyaA translocation assay. The cells were washed twice with RPMI supplemented with 2 mM glutamine and infected with the wild-type strain (JR32) expressing either the 13×*myc*-tagged lpg2552 or lpg1888 at multiplicity of infection of 5. Plates were then centrifuged at 180×*g* for 5 min, incubated at 37°C under CO_2_ (5%) for 1 h, washed 3 times with PBS++ (PBS containing 1 mM CaCl_2_ and 0.125 mM MgCl_2_). The cells were fixed with ice-cold methanol for 5 min, washed twice with PBS and perforated with ice-cold acetone for 2 min. Coverslips were blocked for 10 min with PBS containing 10% BSA and stained with monoclonal chicken anti *myc* antibody (Millipore) diluted 1∶20 and mouse anti *L. pneumophila* antibody (Santa Cruz Biotechnology, Inc.) diluted 1∶100 in PBS containing 10% BSA for 1 h, followed by two 5 min washes in PBS containing 10% BSA. Coverslips were then stained with DAPI (Sigma) and with the secondary antibodies Alexa488 goat anti chicken (Invitrogen Inc) and Cy3 donkey anti mouse (Jackson Immunoresearch Laboratories Inc) diluted 1∶400 in PBS containing 10% BSA, followed by two 5 min washes in PBS containing 10% BSA. Coverslips were then mounted on glass slides using mounting solution (Golden Bridge).

### Imaging, acquisition and processing

Images were acquired using a motorized spinning-disc confocal microscope (Yokogawa CSU-22, Zeiss Axiovert 200 M). The confocal illumination was with 40 mW 473 nm and 10 mW 561 nm solid state lasers. Images were acquired with a 63× oil immersion objective (Plan Apochromat, NA 1.4) For the effectors localization after infection, a Cool Snap HQ-CCD camera (Photometrics) was employed, with a typical exposure times of ∼1 s, images were acquired with 1×1 binning, yielding a pixel size of 0.065 µm. For presentation, fluorescence intensity values were corrected for the contribution of non-specific binding of the secondary/labeled antibody. For the PA and DAG sensors analysis, an Evolve EMCCD camera (Photometrics) was employed, typical exposures of 20–100 ms, 1×1 binning yielding a pixel size of 0.25 µm. Three dimensional image stacks were acquired by sequential acquisition of views recorded every 70–300 ms along the *z*-axis by varying the position of a piezo electrically controlled stage (step size of 0.4 µm). All images were analyzed with SlideBook software (version 5.0; Intelligent Imaging Innovations).

## Supporting Information

Figure S1 The glucose control plates of the experiment presented in Fig. 4.
(PDF)

Figure S2 LecE does not dephosphorylates Pah1.
(PDF)

Figure S3 The glucose control plates of the screen presented in Fig. 8.
(PDF)

Table S1 Plasmids used in this study.
(PDF)

Table S2 Primers used in this study.
(PDF)
