# Supplementary material for: Identification of Two Legionella pneumophila Effectors that Manipulate Host Phospholipids Biosynthesis
Source: PLoS Pathog. 2012 Nov 1;8(11):e1002988. doi: 10.1371/journal.ppat.1002988 (PMC3486869; doi:10.1371/journal.ppat.1002988)
Supplement: Figure S1 — The glucose control plates of the experiment presented in Fig. 4. (PDF) [file ppat.1002988.s001.pdf]

Figure S1

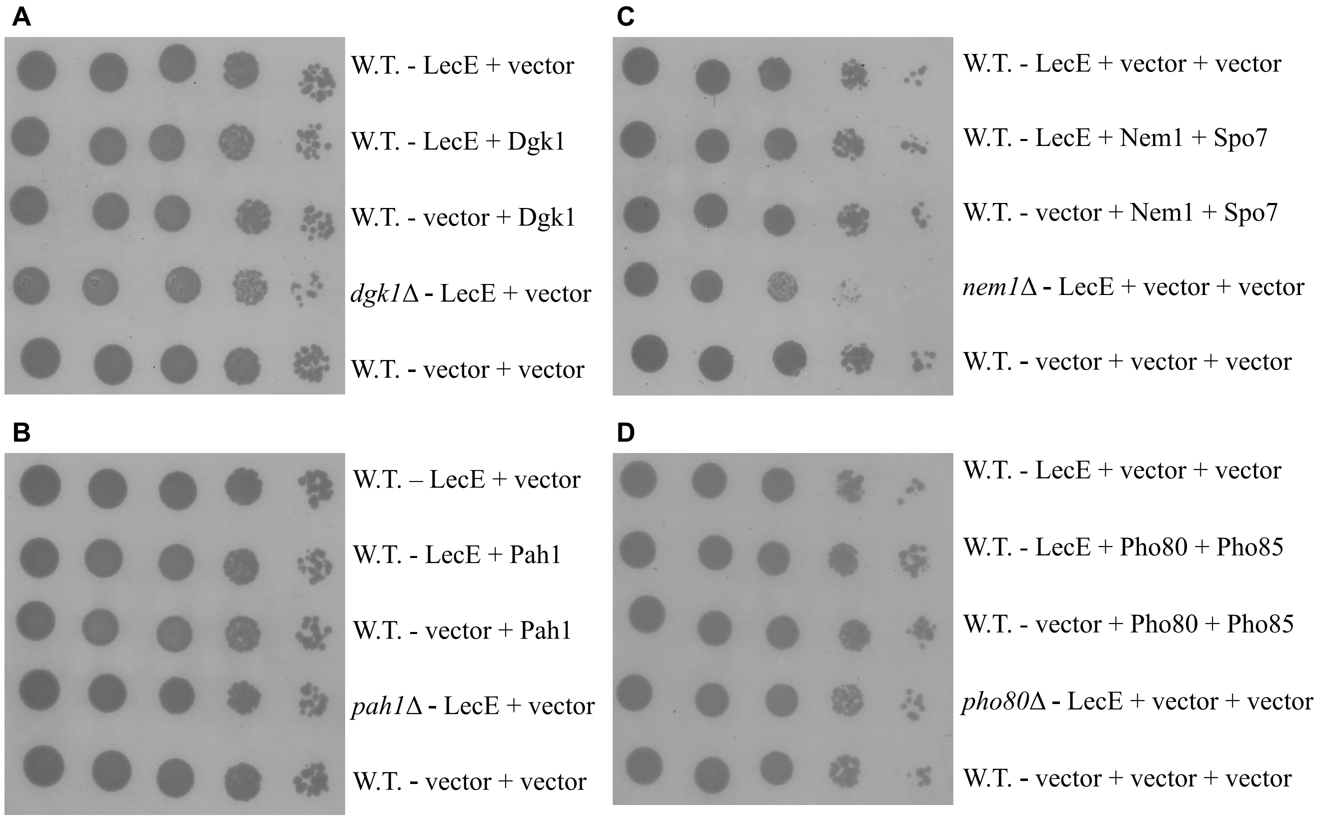

Figure S1. The glucose control plates of the experiment presented in Fig. 4.
LecE was over expressed in a wild-type *S. cerevisiae* BY4741 (W.T.) together with the Dgk1 (A), Pah1 (B), Nem1 and Spo7 (C), or Pho80 and Pho85 (D) genes or in their corresponding deletion mutants: *dgk1*Δ (A), *pah1*Δ (B), *nem1*Δ (C) and *pho80*Δ (D). pGREG523 (vector) was used as a negative control.
