# Supplementary material for: Identification of Two Legionella pneumophila Effectors that Manipulate Host Phospholipids Biosynthesis
Source: PLoS Pathog. 2012 Nov 1;8(11):e1002988. doi: 10.1371/journal.ppat.1002988 (PMC3486869; doi:10.1371/journal.ppat.1002988)
Supplement: Figure S2 — LecE does not dephosphorylates Pah1. (PDF) [file ppat.1002988.s002.pdf]

Figure S2

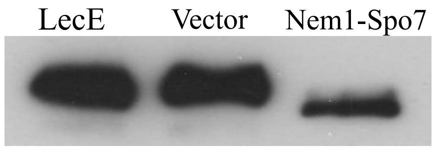

Figure S2. LecE does not dephosphorylates Pah1.
Western blot analysis of HA-tagged Pah1 expressed in wild-type *S. cerevisiae* BY4741 together with LecE, with pGREG523 (vector) or with the Nem1-Spo7 phosphatase complex. Protein levels and size were assessed using α-HA antibody.
