# Supplementary material for: Identification of Two Legionella pneumophila Effectors that Manipulate Host Phospholipids Biosynthesis
Source: PLoS Pathog. 2012 Nov 1;8(11):e1002988. doi: 10.1371/journal.ppat.1002988 (PMC3486869; doi:10.1371/journal.ppat.1002988)
Supplement: Figure S3 — The glucose control plates of the screen presented in Fig. 8. (PDF) [file ppat.1002988.s003.pdf]

Figure S3

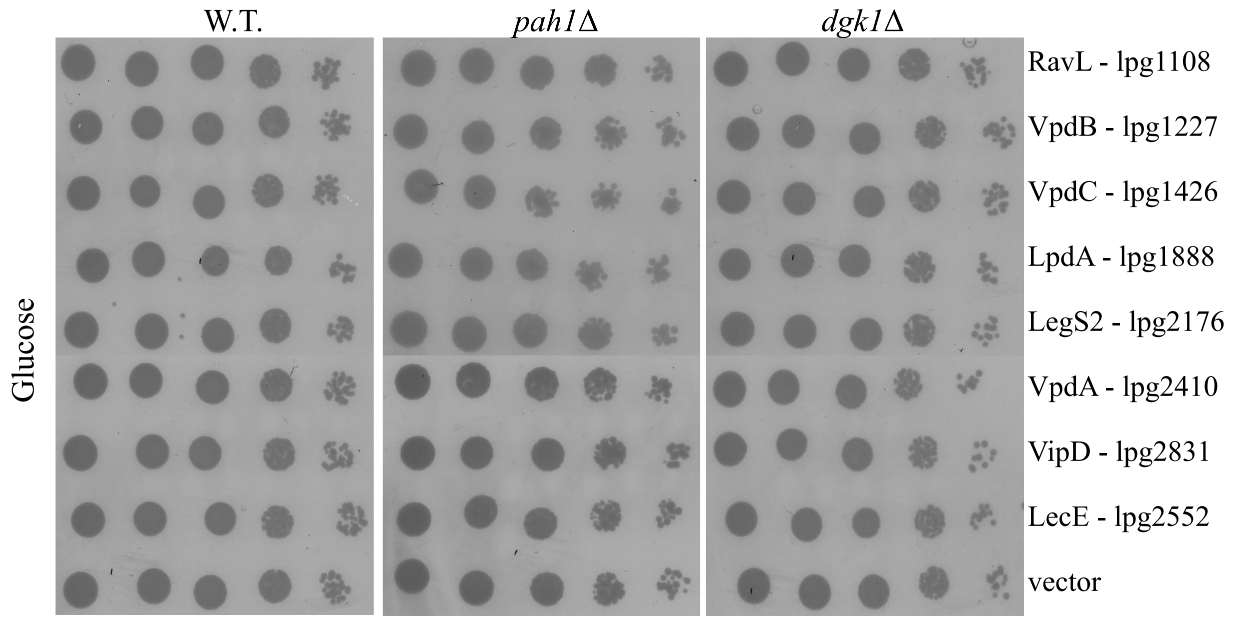

Figure S3. The glucose control plates of the screen presented in Fig. 8.
L. pneumophila effectors (indicated on the right) expected to be involved in or that were shown to function in phospholipids biosynthesis were over-expressed under the GAL1 promoter in wild-type *S. cerevisiae* BY4741 (W.T.), the *pah*1 deletion mutant RV-L8-59 (*pah1*Δ) or in the *dgk*1 deletion mutant (*dgk1*Δ). LecE was used as a positive control. pGREG523 (vector) was used as a negative control.
