# Supplementary material for: Identification of Two Legionella pneumophila Effectors that Manipulate Host Phospholipids Biosynthesis
Source: PLoS Pathog. 2012 Nov 1;8(11):e1002988. doi: 10.1371/journal.ppat.1002988 (PMC3486869; doi:10.1371/journal.ppat.1002988)
Supplement: Table S1 — Plasmids used in this study. (PDF) [file ppat.1002988.s004.pdf]

Table S1 - Plasmids used in this study

Name	Features	Reference or Source
pRV-lpg0581	Full length lpg0581 cloned in pUC-18	This study
pRV-lpg1692	Full length lpg1692 cloned in pUC-18	This study
pRV-lpg1717	Full length lpg1717 cloned in pUC-18	This study
pRV-lpg1887	Full length lpg1887 cloned in pUC-18	This study
pRV-lpg2164	Full length lpg2164 cloned in pUC-18	This study
pRV-lpg2546	Full length lpg2546 cloned in pUC-18	This study
pRV-lpg2552	Full length lpg2552 cloned in pUC-18	This study
pRV-lpg2552-C-ter	C-terminal 92aa of lpg2552 cloned in pUC-18	This study
pRV-lpg1888	Full length lpg1888 cloned in pUC-18	This study
pRV-YlfA	Full length *L. pneumophila ylfA* cloned in pUC-18	This study
pRV-lpg1888-K165R	lpg1888-K165R point mutant cloned in pUC-18	This study
pRV-lpg1888-K376R	lpg1888-K376R point mutant cloned in pUC-18	This study
pRV-VipD	Full length *L. pneumophila vipD* cloned in pUC-18	This study
pRV-VpdA	Full length *L. pneumophila vpdA* cloned in pUC-18	This study
pRV-VpdB	Full leng*th L. pneumophila vpdB* cloned in pUC-18	This study
pRV-VpdC	Full length *L. pneumophila vpdC* cloned in pUC-18	This study
pRV-lpg1108	Full length lpg1108 cloned in pUC-18	This study
pRV-cyaA-lpg0581	lpg0581 fused to CyaA in pMMB-cyaA-C	This study
pRV-cyaA-lpg1692	lpg1692 fused to CyaA in pMMB-cyaA-C	This study
pRV-cyaA-lpg1717	lpg1717 fused to CyaA in pMMB-cyaA-C	This study
pRV-cyaA-lpg1887	lpg1887 fused to CyaA in pMMB-cyaA-C	This study
pRV-cyaA-lpg2164	lpg2164 fused to CyaA in pMMB-cyaA-C	This study
pRV-cyaA-lpg2546	lpg2546 fused to CyaA in pMMB-cyaA-C	This study
pRV-cyaA-lpg2552	lpg2552 fused to CyaA in pMMB-cyaA-C	This study
pRV-cyaA-lpg2552-C-ter	C-terminal 92aa of lpg2552 fused to CyaA in pMMB-cyaA-C	This study
pRV-cyaA-lpg1888	lpg1888 fused to CyaA in pMMB-cyaA-C	This study
pRam-lpg1692	lpg1692 fused to 13x *myc* in pRam	This study
pRam-lpg1717	lpg1717 fused to 13x *myc* in pRam	This study
pRam-lpg2164	lpg2164 fused to 13x *myc* in pRam	This study
pRam-lpg2546	lpg2546 fused to 13x *myc* in pRam	This study
pRam-lpg2552	lpg2552 fused to 13x *myc* in pRam	This study
pRam-YlfA	*L. pneumophila ylfA* fused to 13x *myc* in pRam	This study
pRam-lpg1888	lpg1888 fused to 13x *myc* in pRam	This study
pRam-lpg1888-K165R	lpg1888-K165R point mutant fused to 13x *myc* in pRam	This study
pRam-lpg1888-K376R	lpg1888-K376R point mutant fused to 13x *myc* in pRam	This study
pRam-VipD	*L. pneumophila vipD* fused to 13x *myc* in pRam	This study
pRam-VpdA	*L. pneumophila vpdA* fused to 13x *myc* in pRam	This study
pRam-VpdB	*L. pneumophila vpdB* fused to 13x *myc* in pRam	This study
pRam-VpdC	*L. pneumophila vpdC* fused to 13x *myc* in pRam	This study
pRam-lpg1108	lpg1108 fused to 13x *myc* in pRam	This study
pED-ySPL	*L. pneumophila LegS*2 fused to 13x *myc* in pRam	[1]
pRV-lpg2552-UP	1-kb upstream to the lpg2552 gene in pUC18	This study
pRV-lpg2552-DW	1-kb downstream to the lpg2552 gene in pUC18	This study
pRV-lpg2552-KM	The lpg1888 upstream and downstream regions with the Km cassette between them in pUC-18	This study
pRV-lpg2552::KM-del	The insert of pRV-2552-KM in pLAW344	This study
pRV-lpg1888-UP	1-kb upstream to the lpg1888 gene in pUC18	This study
pRV-lpg1888-DW	1-kb downstream to the lpg1888 gene in pUC18	This study
pRV-lpg1888-GM	The lpg1888 upstream and downstream regions with the Gm cassette between them in pUC-18	This study
pRV-lpg1888::GM-del	The insert of pRV-1888-GM in pLAW344	This study
pGREG536-1888	Lpg1888 fused to 7xHA in pGREG536	This study
pGREG536-1888-K165R	Lpg1888-K165R mutant fused to 7xHA in pGREG536	This study
pGREG536-1888-K376R	Lpg1888-K376R mutant fused to 7xHA in pGREG536	This study
pGREG506-Dgk1	*S. cerevisiae dgk1* in pGREG506	This study
pGREG506-Dgk1-R76A	*S. cerevisiae dgk1*-R76A mutant in pGREG506	This study

pGREG506-Dgk1-D177A	*S. cerevisiae dgk1*-D177A mutant in pGREG506	This study
pSup-13	*S. cerevisiae dgk1, rpl20B, sps4, sfg1, cot1, faa1*-partial gene in pYep24	This study
pSup-13-sub-clone 1	*S. cerevisiae dgk1, rpl20B, sps4*, in pYep24	This study
pSup-13-sub-clone 2	*S. cerevisiae dgk1, rpl20B, faa1*-partial gene in pYep24	This study
pSup-13-sub-clone 3	*S. cerevisiae, sps4*, in pYep24	This study
pGREG503-Spo7	*S. cerevisiae spo7* in pGREG523	This study
pGREG505-Spo7	*S. cerevisiae spo7* in pGREG505	This study
pGREG506-Nem1	*S. cerevisiae nem1* in pGREG506	This study
pGREG505-Nem1	*S. cerevisiae nem1* in pGREG505	This study
pGREG506-Pho85	*S. cerevisiae pho*85 in pGREG506	This study
pGREG504-Pho80	*S. cerevisiae pho*80 in pGREG504	This study
pGREG505-Pho80	*S. cerevisiae pho*80 in pGREG505	This study
pGREG505-Pah1-3xHA	C-terminal fusion of 3xHA with *S. cerevisiae pah1* under its native promoter in promoter-less pGREG505	This study
pGREG505-Pah1-D398E-3xHA	C-terminal fusion of 3xHA with *S. cerevisiae pah1*-D398E mutant under its native promoter in promoter-less pGREG505	This study
pRV-13x *myc*-lpg2552	lpg2552 fused to N-terminal 13x *myc* in pMMB-13x *myc*	This study
pRV-13x *myc*-lpg1888	lpg1888 fused to N-terminal 13x *myc* in pMMB-13x *myc*	This study
pUC-18	oriR(colE1) MCS Amp	[2]
pLAW344	oriR(colE1) sacB oriT(RK2) Cm Amp	[3]
pMMB-cyaA-C	cyaA cloning vector for C-terminal fusions	[4]
pMMB207-NdeI	pMMB207 with an NdeI site between the *pTac* and EcoRI	[4]
pMMB-13x *myc*	pMMB207-NdeI containing the pUC18 polylinker "in-frame" with the13x *myc* tag	This study
pGREG523	*CEN/HIS3/pGAL1/13x myc* cloning vector for C-terminal fusions	[5]
pGERG536	*CEN/URA3/pGAL1/7xHA* cloning vector for C-terminal fusions	[5]
pGERG503	*CEN/HIS3/ pGAL1* cloning vector	[5]
pGERG504	*CEN/TRP1/ pGAL1* cloning vector	[5]
pGERG505	*CEN/LEU2/ pGAL1* cloning vector	[5]
pGERG506	*CEN/URA3/pGAL1* cloning vector	[5]
pYep24	*2μ/URA3* cloning vector in which the yeast genomic library is cloned	[6]
pRam	pGREG523 containing the pUC18 polylinker "in-frame" with the 13x *myc* tag	This study
pRV-mCherry-lpg2552	Lpg2552 fused to mCherry in pmCherryC1	This study
pRV-mCherry-lpg1888	Lpg1888 fused to mCherry in pmCherryC1	This study
pRV-mCherry-lpg1888-K165R	Lpg1888-K165R mutant fused to mCherry in pmCherryC1	This study
pEGFP-Spo20-51-91	*S. cerevisiae spo*20-51-91 fused to GFP in pEGFP-C1	[7]
pEGFP-C1-PKCα	C1 domain of PKCα fused to GFP in pEGFP-C2	[8]
pGFP-KDELR	KDEL receptor fused to GFP	[9]

References

1. Degtyar E, Zusman T, Ehrlich M, Segal G (2009) A *Legionella* effector acquired from protozoa is involved in sphingolipids metabolism and is targeted to the host cell mitochondria. Cell Microbiol 11: 1219-1135.
2. Yanish-Perron, C., J. Viera, and J. Messing (1985) Improved M13 phage cloning vectors and host strains: nucleotide sequences of the M13mp18 and pUC19 vectors. Gene 33:103-119.
3. Wiater, L. A., A. B. Sadosky, and H. A. Shuman (1994) Mutagenesis of *Legionella pneumophila* using Tn903dII*lacZ*: identification of a growth phase-regulated pigmentation gene. Mol. Microbiol. 11:641-653.
4. Zusman T, Aloni G, Halperin E, Kotzer H, Degtyar E, et al. (2007) The response regulator PmrA is a major regulator of the *icm/dot* type IV secretion system in *Legionella pneumophila* and *Coxiella burnetii*. Mol Microbiol 63: 1508-1523.
5. Jansen G, Wu C, Schade B, Thomas DY, Whiteway M (2005) Drag&Drop cloning in yeast. Gene 344: 43-51.
6. Carlson M, Botstein D (1982) Two differentially regulated mRNAs with different 5' ends encode secreted with intracellular forms of yeast invertase. Cell 28: 145-154.
7. Kind gift from Prof. U. Ashery, Department of Neurobiology, Tel-Aviv University
8. Kind gift from Prof. M.G Kazanietz, School of Medicine, University of Pennsylvania
9. Kind gift from Prof. G.Z Lederkremer, Department of Cell Research and ImmunologyTel-Aviv University
