# Supplementary material for: Identification of Two Legionella pneumophila Effectors that Manipulate Host Phospholipids Biosynthesis
Source: PLoS Pathog. 2012 Nov 1;8(11):e1002988. doi: 10.1371/journal.ppat.1002988 (PMC3486869; doi:10.1371/journal.ppat.1002988)
Supplement: Table S2 — Primers used in this study. (PDF) [file ppat.1002988.s005.pdf]

Table S2 - Primers used in this study

Primer name	Sequence (5'-3')
cya-lpg0581F	GCACGAATTCAATGAACACAGATGATATAAATAAA
cya-lpg0581R	GAGCGGATCCTTAAAACTCTTCCCAAATTTC
cya-lpg1692F	GAGCGAATTCAATGGCACGCAGCAAAGAAGA
cya-lpg1692R	GAGCGGATCCCTAACTCGGACCACTCGTACGTAA
cya-lpg1717F	GAGCGAGCTCAATGCCAGTTCGTTATTCAGG
cya-lpg1717R	GACGGTCGACTTAAATGGCCAATGCAGTAGAA
cya-lpg1887F	GAGCGAATTCAATGAAAAAAGTGATATTGTATGC
cya-lpg1887R	GAGCGGATCCTATTGGTTAGCCTTTTGCAAT
cya-lpg2164F	AGCGAATTCAATGTATTTTTCAAGATGTATTTCAC
cya-lpg2164R	GAGCGGATCCGTCACTTATTCCTGCGCTTG
cya-lpg2546F	GACAGAGCTCAATGAGCATAACAGTTAAATCCT
cya-lpg2546R	GAGCGGATCCTTATAAGTTTAGAGTTCTTGAAAC
cya-lpg2552F	AGCGAATTCAATGAAAGAAATAGTTAGTTTATTAG
cya-lpg2552R	GAGTGGATCCGGTTGCTTTAAAAACCTCAT
lpg2552-C-ter-92F	GAGCGAATTCAACCTGGCCCACCGTTTTTGA
lpg1888F	GAGCGAATTCAATGCTCCAATTTTTTGAGAAAG
lpg1888R	GAGCGTCGACAGCCTAAAATCAAGAACAAAC
lpg1888-K165R-F	GTCCAACCATAATAGAGCCATCTGGATTGATGGGG
lpg1888-K165R-R	ATCCAGATGGCTCTATTATGGTTGGACGCAATGGA
lpg1888-K376R-F	GGTTATTCATATGAGATTTATGTCAATTGATGGGC
lpg1888-K376R-R	GACATAAATCTCATATGAATAACCTCTTTTTTCAT
ylfA-cyaA-EcoRI	GAGCGAATTCGATGGCTACTAATGAAACAGAGC
ylfA-cyaA-PstI	GACGCTGCAGCCTTAAATCCTTCTCTTAATTGAC
lpg1108F	GAGCGAATTCAATGTTGAAGCGCGTTCTTAATAC
lpg1108R	GAGAGTCGACGTTTATGGATTGAAAAGG
lpg1227F	AGCGAATTCAATGAAAACGGTAAATAGTCAAAATG
lpg1227R	GAGCGTCGACTTTTACGCCCTATATAGGGATG
lpg2410F	GAGCGGATCCAATGAAAACAAAGCAAGAAGTTTC
lpg2410R	GAGAGTCGACGCTTTGGATTTAAACCTG
Lpg1426-EcoRI	GAGAGAATTCGATGACCCCACAGCACGTC
Lpg1426-BamHI	GACCGGATCCATGTGATGCAAACTCATACG
VipD-F	GAGCGGATCCAATGACAAAAAGCCGTAAATTA
VipD-R	GAGAGTCGACGAAAAATGTTCCGGAATTATTC
2552-up-BamHI	GACAGGATCCGGACAATGTAAATAAACAAATAAG
2552-up-SalI	GACAGTCGACTATTTCTTTCATTACAATAAATCCT
2552-dw-SalI	GACAGTCGACCATAAGTGATGAGGTTTTTAAAGC
2552-dw-EcoRI	GACAGAATTCCAAGCATCAAGTTCATTTTTC
1888-Up-BamHI	CGTCGGATCCCCGGGCGAACTGCAGTCACTGTC
1888-up-EcoRV	GACAGATATCAAAAAATTGGAGCATGATTC
1888-DW- EcoRV	GACAGATATCTGTTTTGTTTGTTCTTGATTTTAG
1888-DW- EcoRI	GACAGAATTCCCGGGAAAATGTAGCATATCAATGC
DGK1-SpeI	GAGCACTAGTATGGGGACCGAAGATGCCA
DGK1-SalI	GAGCGTCGACATGCTTTTATTTCTTGAATGTTTTG
DGK-R76A-F	CATGAAATTCCCGCTAAGGTGTTCCATTCTTCCAT
DGK-R76A-R	CACCTTAGCGGGAATTTCATGTTTAGTTATAAAAT
DGK-D177A-F	CTAAGTTGGTCCGCTACAGCCGCCGCAACTATTG
DGK-D177A-R	GCGGCTGTAGCGGACCAACTTAGCAAAAATAACG
Cdc28-SpeI	GAGCACTAGTATGAGCGGTGAATTAGCAAATTAC
Cdc28-SalI	GAGCGTCGACTTATGATTCTTGGAAGTAGGGGTG
NEM1-SpeI	GTGCACTAGTATGAATGCCCTAAAATATTTCTC
NEM1-SalI	GAGCGTCGACAATTGTCAGTTTATGTTGAATG
SPO7-SpeI	GTGCACTAGTATGGAGCCAGAGAGCATAGG
SPO7-SalI	GAGCGTCGACTCTTTCATTCTGATTTAGGTCG

Pah1-for	GCTCCAGCTGCTCTCTTCGTTCGCAGTTCC
Pah1-rev	GTTATCTAGAATCTTCGAATTCATCTTCGTC
Pah1-D398E-for	GTTATCAGTGAAATTGATGGCACCATCACAAAATC
Pah1-D398E-rev	GCCATCAATTTCACTGATAACAATTGGAACATCCC
HA-For	GCTATCTAGAGGAATCTTTTACCCATACGATGT
HA-Rev	GCTCGTCGACCTAGCACTGAGCAGCGTAATCTG
Pah1-kanMX-for	ATGCAGTACGTAGGCAGAGCTCTTGGGTCTGTGTCTAAAACATGGTCTTCATCGATGAATTCGAGCTC
Pah1-kanMX-rev	TTAATCTTCGAATTCATCTTCGTCGAATTCATCGTCATCGAATTCATCGCGTACGCTGCAGGTCGAC
NEM1-kanMX-for	ATGAATGCCCTAAAATATTTCTCAAATCATTTAATAACTACAAAGAAACAATCGATGAATTCGAGCTC
NEM1-kanMX-rev	TCAGTTTATGTTGAATGCCTTCTCTCCATGTTTCAATGCCAGTATATTCCGTACGCTGCAGGTCGAC
Myc-F-NdeI	GACACATATGATCCCCGGGTTAATTAACGG
Myc-R-yeast	AGGACCGTGCAGCGGATCCA
Pho85-SalI-for	GAGCGTCGACAATGTCTTCTTCTTCACAGTATG
Pho85-SalI-rev	GAGCGTCGACGGGCTGCGTTATGAAGC
Pho80-SpeI	GTGCACTAGTATGGAAAGCACATCAGGAGAAC
Pho80-SalI	GAGCGTCGACGAAAAATCATTAATCTGGCTTTG
